# A commented checklist and key for the genus *Carex* (Cyperaceae) in Peru

**DOI:** 10.3897/phytokeys.271.178418

**Published:** 2026-02-26

**Authors:** Pedro Jiménez-Mejías, Pablo García-Moro, Andrea A. Camilo, Piero M. Mazzei, Marcial Escudero, Santiago Martín-Bravo, Asunción Cano

**Affiliations:** 1 Área de Botánica, Departamento de Biología Molecular e Ingeniería Bioquímica, Universidad Pablo de Olavide, Sevilla, Spain Museo de Historia Natural, Universidad Nacional Mayor de San Marcos Lima Peru; 2 Laboratorio de Florística, Departamento de Dicotiledóneas, Museo de Historia Natural, Universidad Nacional Mayor de San Marcos, Av. Arenales 1256, Lima 11, Peru Facultad de Ciencias Biológicas, Universidad Nacional Mayor de San Marcos Lima Peru; 3 Department of Plant Biology and Ecology, University of Sevilla, Sevilla, Spain Área de Botánica, Departamento de Biología Molecular e Ingeniería Bioquímica, Universidad Pablo de Olavide Sevilla Spain; 4 Instituto de Investigación de Ciencias Biológicas, Facultad de Ciencias Biológicas, Universidad Nacional Mayor de San Marcos, Av. Venzuela, s/n, Lima 1, Peru Department of Plant Biology and Ecology, University of Sevilla Seville Spain

**Keywords:** Andes, bofedal, Cordillera, sedges, *
Uncinia
*

## Abstract

*Carex* is one of the most species-rich genera of vascular plants worldwide and it is a dominant element in cold and temperate regions. However, its diversity in South America is still less understood than in other areas of the World. In Peru, the genus is a key component of high Andean grasslands and wetlands, yet the last comprehensive taxonomic treatment dates back to 1936 when only 18 species were recognised. Subsequent checklists increased the number to 25, but current estimates remained incomplete and largely based on outdated identifications. Here, we present a taxonomic update of *Carex* in Peru, based on extensive fieldwork, herbarium studies across major Peruvian and international collections, literature review and complementary records from citizen-science platforms. Our study documents 56 species, including six strict endemics and three subendemics, with Cajamarca emerging as the richest Department (29 species). Four species are here confirmed for Peru for the first time. We provide an identification key in English and Spanish and updated distribution maps for all species, as well as a preliminary conservation assessment for 24 rare or endemic taxa using IUCN Criteria. Despite its ecological relevance, *Carex* remains understudied in Peru, with many species overlooked due to collecting biases, inconspicuous morphology and geographic barriers. Our findings call for integrative taxonomic, ecological and conservation studies to improve the knowledge on Andean *Carex* and secure their long-term preservation under accelerating climate change.

## Abstract (es)

*Carex* es uno de los géneros de plantas vasculares más ricos en especies a nivel mundial y constituye un elemento dominante en regiones frías y templadas. Sin embargo, su diversidad en Sudamérica sigue siendo menos conocida que en otras regiones del mundo. En Perú, el género es un componente clave de los pastizales y humedales altoandinos, aunque el último tratamiento taxonómico exhaustivo se remonta al trabajo de Macbride de 1936, en el que se reconocieron tan solo 18 especies. Listados posteriores han incrementado este número hasta 25, pero las estimaciones actuales permanecen incompletas y basadas en parte en identificaciones obsoletas. En este trabajo presentamos una actualización taxonómica del género *Carex* en Perú basada en un amplio trabajo de campo y el estudio de herbarios de las principales colecciones peruanas e internacionales, así como la revisión bibliográfica y registros complementarios procedentes de plataformas de ciencia ciudadana. Nuestro estudio documenta 56 especies, incluidas seis endémicas estrictas y tres subendémicas, siendo Cajamarca el departamento con mayor riqueza específica (29 especies). Cuatro especies se confirman aquí por primera vez para Perú. Proporcionamos una clave de identificación en inglés y español y mapas de distribución actualizados para todas las especies, así como una evaluación preliminar del estado de conservación de 24 taxones raros o endémicos siguiendo los criterios de la UICN. A pesar de su relevancia ecológica, *Carex* sigue siendo un género poco estudiado en Perú, con numerosas especies pasadas por alto debido a sesgos de muestreo, su morfología poco conspicua y las barreras geográficas. Nuestros resultados ponen de manifiesto la necesidad de estudios integradores de carácter taxonómico, ecológico y de conservación para mejorar el conocimiento de las *Carex* andinas y asegurar su preservación a largo plazo frente al acelerado cambio climático.

## Introduction

With more than 2000 spp, *Carex* L. (Cyperaceae) is one of the five most diverse vascular plant genera worldwide (Roalson et al. 2021; POWO 2025). The genus is also the most diverse amongst the Cyperaceae (the sedge family), which is, in turn, one of the ten largest plant families. Unlike most sedges, *Carex* has a clear preference for cold and temperate regions, being absent or showing very poor species richness in Tropical lowland rainforests and in hot deserts. It has primarily diversified in the Northern Hemisphere and it has dispersed multiple times to the South (Martín-Bravo et al. 2019). In the Tropics, the genus thrives primarily in mountain regions and is present in all the major ranges in that region, including the Andes.

**Figure 1.**
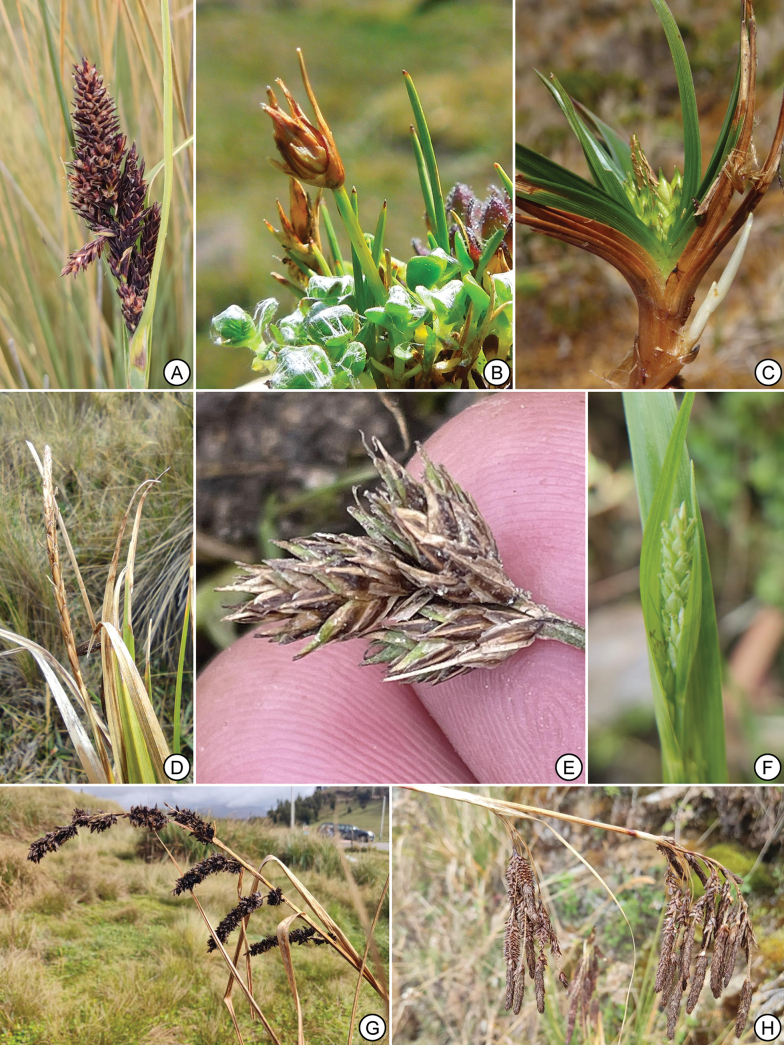
Representative living specimens of Peruvian *Carex* species. **A**. *Carex canoi* (Cajamarca); **B**. *Carex herba-alpacae* (Lima); **C**. *Carex paramorum* (Colombia, Cauca); **D**. *Carex laegaardii* (Áncash); **E**. *Carex mandoniana* (Lima); **F**. *Carex roalsoniana* (Piura); **G**. *Carex david-smithii* (Cajamarca); **H**. *C. hebetata* (Áncash). Credits: P. Jiménez-Mejías (A,F,G), J.J. Alegría (B), J. Calvo (C), S. Martín-Bravo (D,H) and L. Vilca Bustamante (E, taken from iNaturalist).

In Peru, *Carex* is an important element in high Andean tall grasslands and wetlands (locally known as pajonales and bofedales, respectively), although species can also be found in dry high mountain steppes (puna), and montane forests (Fig. 1). Despite its high diversity and ecological importance, the only taxonomic account of the genus in Peru is the revision for the Flora of Peru by Macbride (1936) and which only included 18 *Carex* species (including those formerly placed in *Uncinia* Pers.). Later, Brako and Zarucchi’s (1993) checklist would increase the number of accepted species to 25 (with a total of 28 taxa), containing several names without confirming vouchers, and Ulloa Ulloa et al. (2018 onwards) 28 species. As of today, POWO (2025) reports up to 46 *Carex* species for Peru. However, since the majority of identifications in herbaria have been based on Macbride`s (1936) work, most of the *Carex* diversity in Peru has remained unnoticed to date. This situation is parallel to what happens in the rest of South America, with most *Carex* groups insufficiently studied and only a few recent integrative revisions or regional treatments available (Jiménez-Mejías et al. 2021a; Muñoz-Schüler et al. 2023; Morales-Alonso et al. 2024a; Lois et al. 2025). Consequently, new species are continuously discovered (e.g. Jiménez-Mejías and Escudero (2016); Muñoz-Schüler et al. (2024); González-Gallego et al. (2025)) and relevant new chorological records have been made (e.g. Jiménez-Mejías et al. (2018, 2020a); Muñoz-Schüler et al. in press) during the last years.

After years of taxonomic work including herbarium revisions, literature search and fieldwork, we present in this study an updated checklist and identification key for the genus *Carex* in Peru.

## Material and methods

### Sampling

A 38-days fieldwork campaign in Peru in 2021 was performed along the Andes, spanning from Puno to Piura, by P.J.-M., P.G.-M. and S.M.-B. A full revision of the Peruvian Herbaria CPUN, HSP, HUSA, HUT, MOL, MOLF and USM, as well as of the Peruvian collections of the North American Herbarium F, was performed by M.E. with double check of detailed images, as well as specimens at USM, by P.J.-M. The full collections of Neotropical *Carex* at A, GH, K, MO, NY and US were studied by P.J.-M. Type material was consulted in all the instances to ensure the proper use of the names.

Photography-based observations in iNaturalist (www.inaturalist.org) were considered when proper identification was possible. This citizen-science platform allows the user to upload georeferenced photos of organisms and make them available for consultation to researchers. iNaturalist observations are growingly used in research (e.g. Henning et al. (2023)), although given some limitations, these data should be considered with caution (e.g. Koo et al. (2022); López-Guillén et al. (2024); McMullin and Allen (2022)), especially for taxonomically difficult groups such as *Carex*, in which key diagnostic characters may not be clearly visible, rendering the identification in the observation potentially ambiguous. All *Carex* photos georeferenced in Peru available until March 2025 (a total of 105 observations) were checked by P.J.-M. (iNaturalist user @pjimmej). Whenever possible, the taxonomic identity of the plants in the photos was provided within the platform.

### Taxonomic and chorological revision

For the initial identification of the material, we used the keys provided in Kükenthal (1909), Macbride (1936), Muñoz-Schüler et al. (2023, in press), Morales-Alonso et al. (2025), Lois et al. (2025) and González-Gallego et al. (2025), as well as comparison with type material. An identification key for the resulting 56 *Carex* taxa for the flora of Peru (see results) was built from the available keys and primary observations on herbarium material by the first author (P.J.-M.), incorporating revisions by the other authors of this paper. Additional relevant specialised literature was consulted for systematics, taxonomic and chorological data. Each of these publications is cited under the corresponding species epigraph.

New or relevant chorological records were identified with respect to the information reported in Brako and Zarucchi (1993) and POWO (2025) for Peru. Distributions reported for South America and/or the rest of the World are based either on Plants of the World Online checklist (POWO 2025) or the online treatments of www.cyperaceae.org (Jiménez-Mejías et al. 2025). To avoid redundancy and ease the reading of this study, we will avoid continuously citing these works unless necessary. If additional distributional data from alternative sources are used, these are listed under the corresponding species epigraph.

All the available distribution data from herbarium specimens, field collections and iNaturalist observations were compiled and plotted on a regional map of Peru using the ‘terra’ package (Hijmans 2025) in RStudio (R Core Team 2025).

### Preliminary conservation assessment

Amongst the *Carex* species present in Peru, we selected 24 species of conservation interest due to their endemicity and/or rarity and conducted a preliminary, informal evaluation of their conservation status at the national level using IUCN Red List categories, criteria and guidelines (IUCN Standards and Petitions Committee 2024). We are aware that information on the conservation status of *Carex* species in Peru is very limited (see Discussion). To obtain a preliminary tentative assessment for the selected species, this was based on criterion B (geographic range based on known occurrences). We evaluated the potential threat category that could apply according to geographic range thresholds, even though a full application of criterion B was not possible due to the lack of data on the specific conditions required to meet it. This “better safe than sorry” approach should be regarded only as a first step towards a more accurate understanding of the conservation status of *Carex* in Peru, which is currently almost unknown.

**Figure 2.**
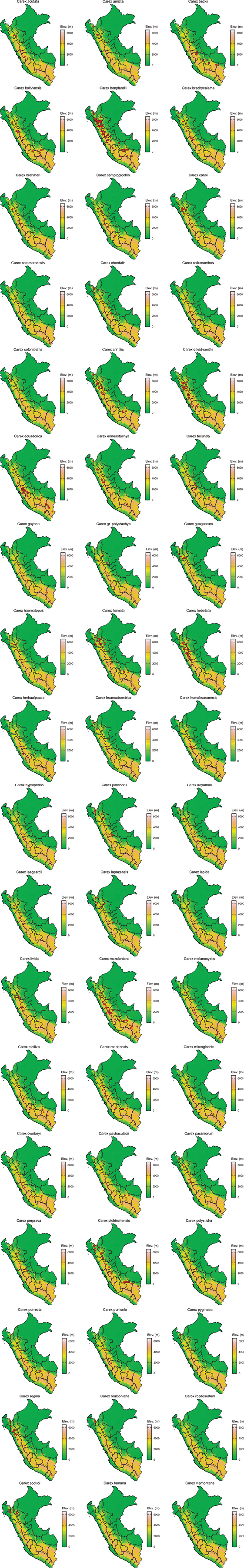
Known distribution of *Carex* species in Peru according to the studied herbarium records, field collections and reliable iNaturalists observations.

Range descriptors ‘extent of occurrence’ (EOO) and ‘area of occupancy’ (AOO), used for the application of IUCN criterion B, were calculated (2 km cell width) for the Peruvian ranges of the selected species using the GeoCAT tool (see https://geocat.iucnredlist.org/; Bachman et al. (2011)) based on the coordinates of all known Peruvian studied material (see Results, Fig. 2). For species with occurrences reduced to 1–2 grid cells, EOO cannot be calculated. Species occurrences were quantified applying the IUCN concepts of ‘location’ (based on the potential threats affecting all taxon individuals in an area) and ‘subpopulation’ (based on limited genetic exchange) (IUCN Standards and Petitions Committee 2024). A layer with the protected areas (World Database on Protected Areas 2017), superimposed on the species range in GeoCAT, allowed us to check if occurrences were on protected land.

## Results and discussion

### Update on the diversity of *Carex* in Peru

To date, 46 species of *Carex* (including the species formerly placed in *Uncinia*) have been reported from Peru (POWO 2025). After our revision, the total number of accepted species has increased to 56, all of them native, present in 18 Departments (Tables 1, 2, Fig. 2, Suppl. material S1, S2). The Department with the least species confirmed is Tacna (one species) and the most diverse one is Cajamarca (29 species).

The most broadly distributed taxon is *C. bonplandii* s.l. (present in 14 Departments), followed by *C. mandoniana* and *C. pichinchensis* (each in 11 Departments) (Table 2). Six species are strict endemics from Peru: *Carex canoi* (associated with the northern Huancabamba Region; see below), *C. david-smithii*, *C. hebetata* and *C. hypsipedos* (all them from the high elevations of Central Peru), *C. pachacutecii* (from the eastern valleys of S Peru) and *C. ros-desertum* (endemic to the coastal hills ("lomas") of Arequipa and Tacna). To these, three subendemic species should be added: *C. regina*, from C and N Peru, occurring marginally in Ecuador, *C. roalsoniana*, from the Huancabamba Region, also reaching southern Ecuador and *C. herba-alpacae*, an altiplano species that marginally enters Bolivia.

**Table 1.** List of *Carex* species reported from Peru and its taxonomic and chorologic update regarding previously published sources. Names originally listed in Macbride (1936), Brako and Zarucchi (1993) and POWO (2025) are respectively marked with the superindices ^1^, ^2^ and ^3^. Five species from adjacent territories not known in Peru (marked with an *) are included as their presence seems possible. Taxonomic groups above species rank follow Roalson et al. (2021) with minor modifications according to Jiménez-Mejías et al. (2025). For pertinent explanations on the particular cases, the reader is referred to the main text.

Taxon name	Confirmed/Absent, endemic?	Changes implemented in this work
**A. Subg. *Psyllophorae***		
**A.1. Sect. *Junciformes***		
*Carex camptoglochin* V.I.Krecz.^3^ (≡ *C. microglochin* var. *fuegina* Kük.^2^)	Confirmed	
*Carex herba-alpacae* Jim.Mejías & A.Mor.Alonso	Confirmed, subendemic	
*Carex melliza* A.Mor.Alonso, Donadío & Jim.Mejías	Confirmed	Previously included in *C. phalaroides*.
*Carex phalaroides* Kunth	Absent	Records now included in *C. melliza* and *C. viamontana*.
*Carex setifolia* Kunze^1,2,3^	Unconfirmed; revision needed.	
*Carex vallis-pulchrae* Phil.^3^	Absent	Records now recognized as *C. herba-alpacae*.
*Carex viamontana* A.Mor.Alonso & Jim.Mejías	Confirmed	Previously included in *C. phalaroides*.
**B. Subg. *Euthyceras***		
**B.1. Capitata clade**		
*Carex microglochin* Wahlenb.^2,3^	Confirmed	
**C. Subg. *Vignea***		
**C.1. Annectens clade**		
*Carex ecuadorica* Kük. ^1,2,3^	Confirmed	
*Carex gayana* É.Desv. ^2,3^	Confirmed	
*Carex ownbeyi* G.A.Wheeler^3^	Confirmed	
*Carex praegracilis* W.Boott^1,2^	Absent	The record corresponds to *C. ecuadorica*.
**C.2. Sect. *Cyperoideae***		
*Carex bonplandii* Kunth. ^1,2,3^	Confirmed	
*Carex macloviana* D’Urv.^1,2,3^	Absent	The records probably correspond to *C. mandoniana* or *C. ecuadorica*.
*Carex mandoniana* Boeckeler^1,2,3^	Confirmed	
*Carex peucophila* Holm^3^	Absent	The name has been used in herbaria to refer to plants here treated under *C. bonplandii*. The published record corresponds to *C. mandoniana*.
**C.3. Sect. *Bracteosellae***		
*Carex bonariensis* Desf.^2,3^	Absent	Records now included in *C. guaguarum* and *C. ros-desertum*.
*Carex guaguarum* Jim.Mejías & Muñoz-Schüler	Confirmed	Previously included in *C. bonariensis*.
*Carex ros-desertum* Jim.Mejías & Muñoz-Schüler	Confirmed, endemic	Previously included in *C. bonariensis*.
**C.4. Disticha clade**		
*Carex maritima* Gunnerus	Absent	Records now recognised as *C. melanocystis*.
*Carex melanocystis* É.Desv.^3^	Confirmed	
**D. Subg. *Uncinia***		
**D.1. Sect. *Wheelerianae***		
*Carex lepida* Boott^3^	Confirmed	
*Carex pachamamae* Jim.Mejías & Reznicek*	Unconfirmed; presence probable	
*Carex roalsoniana* Jim.Mejías & M.Escudero^3^	Confirmed	
**D.2. Sect. *Uncinia***		
*Carex brevicaulis* Thouars^3^ (≡*U. brevicaulis* Thouars^2^)	Absent	The published record corresponds to *C. laegaardii*
*Carex erinacea* Cav. (≡ *U. erinacea* (Cav.) Pers.; *= U. longifolia* Kunth)	Absent	Report probably erroneous.
*Carex goetghebeurii* J.R.Starr* (≡ *U. tenuifolia* G.A.Wheeler & Goetgh.)	Unconfirmed; presence probable	
*Carex hamata* Sw.^3^ (≡*U. hamata* (Sw.) Urb.^1,2^)	Confirmed	
*Carex laegaardii* J.R.Starr^3^ (≡*U. paludosa* G.A.Wheeler & Goetgh.)	Confirmed	
*Carex meridensis* (Steyerm.) J.R.Starr ^3^ (=*U. macrolepis* Decne)	Confirmed	
*Carex koyamae* (Gómez-Laur.) J.R.Starr (≡*U. koyamae* Gómez-Laur.)	Confirmed	
*Carex phleoides* Cav.^3^ (≡ *U. phleoides* (Cav.) Pers.^1,2^)	Absent	Records now recognised as *C. koyamae*.
**E. Subg. *Carex***		
**E.1. Decora clade**		
*Carex amicta* Boott^3^	Confirmed	
*Carex beckii* G.A.Wheeler	Confirmed	Previously included in *C. polystachya*.
*Carex polystachya* Wahl^2,3^ (=*C. cladostachya*^1^ auct. non Wahl)	Absent	Records now included in *C. beckii* and *C. sodiroi*.
*Carex porrecta* Reznicek & Camelb.	Confirmed	
*Carex sodiroi* Kük.	Confirmed	Previously included in *C. polystachya*.
**E.2. Sect. *Abditispicae***		
*Carex collumanthus* (Steyerm.) L.E.Mora^3^	Confirmed	
*Carex humahuacaensis* G.A.Wheeler^3^	Confirmed	
*Carex ruthsatzae* G.A.Wheeler*^3^	Unconfirmed, presence probable	
**E.3. Sect. *Paniceae***		
*Carex brachycalama* Griseb.^1,2,3^	Confirmed	Cited by POWO, probably based on a Bolivian collection adjacent to the border (Wheeler 2002b). No material seen in this work
*Carex livida* (Wahlenb.) Willd.^3^	Confirmed	
**E.4. Hirta clade**		
*Carex acutata* Boott^1,2,3^	Confirmed	
*Carex haematopus* Jim.Mejías & Roalson^3^	Confirmed	
*Carex hookeri* Kunth^3^ (≡*C. beechyana* Boott^1,2^)	Absent	The records probably correspond to *C. haematopus*.
*Carex paramorum* Jim.Mejías	Confirmed	
*Carex polysticha* Boeckeler^2,3^ (=*C. pseudocyperus*^1^ auct. non L.)	Confirmed	
*Carex tamana* Steyerm.	Confirmed	
*Carex tristicha* Boott, nom. illeg.	–	New legitimate name: *C. paramorum*.
**E.5. Sect. *Racemosae***		
*Carex hypsipedos* C.B.Clarke^3^	Confirmed, endemic	The taxonomic concept of the name used here attaches to that of Morales-Alonso et al. (2024b). Previously the name has been used to refer to *C. punicola*.
*Carex phylloscirpoides* Saldivia, S.Gebauer, Martín-Bravo & Jim.Mejías*	Unconfirmed; presence probable	
**E.6. Sect. *Phacocystis***		
*Carex brehmeri* Boeckeler^3^	Confirmed	
*Carex enneastachya* C.B.Clarke^3^	Confirmed	
**E.7. Sect. *Fecundae***		
*Carex canoi* Jim.Mejías & Escudero	Confirmed, endemic	
*Carex catamarcensis* Kük.	Confirmed	
*Carex colombiana* Jim.Mejías, Lois, Acedo, & A.Cano	Confirmed	
*Carex chordalis* Lieb.^3^ (=*C. jamesonii* var. *gracilis*^1,2^ auct. non LH.Bailey)	Confirmed	
*Carex david-smithii* Reznicek^3^	Endemic	
*Carex fecunda* Steud.^1,2,3^	Confirmed	
*Carex jamesonii* Boott^1,2,3^	Confirmed	
*Carex lapazensis* C.B.Clarke	Confirmed	
*Carex lemanniana* Boott^2,3,^*	Unconfirmed; presence probable.	The published record corresponds to *C. regina*. Other material determined in herbaria matches *C. canoi*.
*Carex pachacutecii* Jim.Mejías, Lois, García-Moro, Martín-Bravo & A. Cano	Confirmed, endemic	
*Carex perprava* C.B.Clarke (= *C. fecunda* var. *atropurpurea* (Boeckeler) J.F.Macbr ^1,2^)	Confirmed	
*Carex pichinchensis* Kunth^1,2,3^	Confirmed	
*Carex regina* Jim.Mejías, Lois, Acedo & A.Cano	Confirmed, subendemic	
**E.8. Castanea clade**		
*Carex boliviensis* Van Heurck & Müll.Arg. ^2,3^	Confirmed	
*Carex crinalis* Boott^1,2,3^	Confirmed	
*Carex hebetata* Boott^1,2,3^	Confirmed, endemic	
*Carex huancabambica* Gonz.Gallego & Jim.Mejías	Confirmed	
*Carex pygmaea* Boeckeler^3^	Confirmed	Here confirmed. The previous report refers to *C. paramorum*.
**E.9. Sect. *Spirostachyae***		
*Carex peruviana* J.Presl. & C.Presl.^3^	Absent	Report erroneous (Muñoz-Schüler et al. in press).
**E.10. Sect. *Acrocystis***		
*Carex punicola* D.B.Poind., Jim.Mejías & M.Escudero^3^	Confirmed	Previously listed as *C. hypsipedos*.
*Carex hypsipedos*^1,2^ auct. non C.B.Clarke	Confirmed	Records before Poindexter et al. (2017) refers to *C. punicola*.

**Table 2.** Checklist of species per Department. Species are presented in alphabetical order and Departments are presented from north to south and, within a same latitude range, from west to east. Confirmed presence is depicted with the symbol “✓”, while species whose occurrences is not confirmed, but possible is indicated with a question mark “?”.

Species	Piura	Cajamarca	Amazonas	Lambayeque	San Martín	La Libertal	Áncash	Huánuco	Lima	Pasco	Junín	Cusco	Huancavelica	Ayacucho	Apurimac	Puno	Arequipa	Moquegua	Tacna	Total departments
*C. acutata*		✓				✓		✓			✓									4
*C. amicta*	✓	✓																		2
*C. beckii*					?	?				✓	✓	✓		✓		✓				5
*C. boliviensis*		✓					✓	✓				✓		✓	✓					6
*C. bonplandii*	✓	✓	✓	✓	✓	✓	✓	✓	✓	✓	✓	✓	✓		✓					14
*C. brachycalama*	✓	✓					✓	✓		✓										5
*C. brehmeri*		✓					✓	✓												3
*C. camptoglochin*		✓			✓		✓	✓	✓		✓		✓							7
*C. canoi*		✓			✓	✓														3
*C. catamarcensis*																	✓		✓	2
*C. chordalis*	✓	✓	✓	✓				✓		✓	✓									7
*C. collumanthus*								✓	✓								✓			3
*C. colombiana*		✓																		1
*C. crinalis*	✓	✓				✓	✓					✓	✓		✓					7
*C. david-smithii*		✓	✓		✓	✓	✓	✓	✓		✓			✓	✓					10
*C. ecuadorica*		✓					✓		✓	✓	✓	✓	✓			✓	✓			9
*C. enneastachya*	✓	✓					✓					✓								4
*C. fecunda*							✓			✓	✓	✓								4
*C. gayana*											✓					✓				2
*C. goetghebeurii*	?																			0
*C. guaguarum*		✓		✓																2
*C. haematopus*		✓																		1
*C. hamata*		✓	✓		✓	✓		✓		✓	✓	✓								8
*C. hebetata*		✓			✓	✓	✓	✓	✓											6
*C. herba-alpacae*									✓				✓				✓			3
*C. huancabambica*	✓	✓																		2
*C. humahuacaensis*									✓	✓										2
*C. hypsipedos*											✓									1
*C. jamesonii*	✓	✓	✓		✓					✓										5
*C. koyamae*	✓	✓						✓			✓	✓			✓	✓				7
*C. laegaardii*	✓	✓					✓													3
*C. lapazensis*		✓	✓		✓					✓										4
*C. lemanniana*	?	?																		2
*C. lepida*	✓																			1
*C. livida*					✓	✓		✓												3
*C. mandoniana*		✓				✓	✓	✓	✓		✓	✓	✓	✓	✓	✓				11
*C. melanocystis*		✓								✓	✓					✓	✓			5
*C. melliza*												✓	✓	✓		✓				4
*C. meridensis*						✓	✓	✓				✓	✓	✓						6
*C. microglochin*								✓	✓			✓								3
*C. ownbeyi*											✓	✓		✓						3
*C. pachacutecii*												✓			✓					2
*C. pachamamae*																?				0
*C. paramorum*	✓	✓				✓	✓	✓			✓									6
*C. perprava*			✓		✓					✓	✓	✓								5
*C. phylloscirpoides*																			?	0
*C. pichinchensis*	✓	✓	✓	✓	✓	✓	✓	✓			✓	✓				✓				11
*C. polysticha*										✓										1
*C. porrecta*											✓	✓								2
*C. punicola*										✓	✓	✓								3
*C. pygmaea*	✓																			1
*C. regina*	✓	✓		✓		✓	✓													5
*C. roalsoniana*	✓																			1
*C. ros-desertum*																	✓	?	?	1
*C. ruthsatzae*																?				0
*C. setifolia*																			?	0
*C. sodiroi*	✓	✓	✓		?	?														3
*C. tamana*												✓								1
*C. tristicha*																				0
*C. via-montana*	✓																			1
Total confirmed species	19	30	9	5	11	13	17	18	10	14	20	20	8	7	7	8	6	0	1	

Ten of these species have been recently described, namely *C. canoi*, *C. colombiana*, *C. guaguarum*, *C. herba-alpacae*, *C. huancabambica*, *C. melliza*, *C. pachacutecii*, *C. regina*, *C. ros-desertum* and *C. via-montana*. *Carex porrecta* and *C. tamana*, both known from N South America, are reported for the first time for Peru in this publication. In addition, *C. tristicha* Boott nom. illeg., also known from N South America, is confirmed for Peru and the taxon renamed as *C. paramorum*.

Several previously reported species need to be placed under different names (see Table 1). The Peruvian records of the boreal *C. maritima* belong to the closely-related *C. melanocystis*, those of *C. phleoides* to *C. koyamae* and those of *C. vallis-pulchrae* to *C. herba-alpacae*. Records of three species need to be split between more than one name: *C. polystachya* reports need to be placed either under *C. beckii* or *C. sodiroi*, those of *C. phalaroides* under *C. melliza* or *C. via-montana* and those of *C. bonariensis* either in *C. guaguarum* or *C. ros-desertum*. The presence of *C. pygmaea* in Peru is confirmed, but the previously known record is invalidated. The records of *C. hookeri* (*= C. beechyana*), *C. brevicaulis*, *C. macloviana*, *C. peruviana* and *C. praegracilis* are dismissed after no evidence of these species was found in herbaria or iNaturalist. The presence of *C. setifolia* in Peru from a previous report, based on an unlocated specimen, is discussed.

Table 1 presents a comprehensive list of the taxa reported from Peru and their regional distribution in the country. Fig. 2 displays the known distribution of the taxa based on herbarium records, field collections and verified iNaturalists observations.

### Biogeography of *Carex* in Peru

The distribution of the different species of *Carex* in Peru reveals a series of biogeographic patterns that somehow mirror the bioregionalisation of the country.

Up to eleven species are found only in the northern Andean regions, which climatically contrast with the rest of the cordillera in Peru mainly because of its more elevated precipitation (Valencia 1992). These are northern Andean species that have their southernmost distribution limit in Peru (*C. amicta*, *C. colombiana*, *C. guaguarum*, *C. haematopus*, *C. huancabambica*, *C. pygmaea*, *C. sodiroi*, *C. via-montana*) or are (sub)endemic elements related to the bioclimatic and orographic transition area of Amotape-Huancabamba (*C. canoi*, *C. roalsoniana*) (Weigend 2002).

Eleven species have distributions related to the mesic montane forests of the Amazonian side of the Andes. Some of these seem to be entirely confined to the eastern half of the Cordillera (*C. beckii*, *C. fecunda*, *C. koyamae*, *C. pachacutecii*, *C. perprava*, *C. porrecta*), while others become more widespread through the wet northern regions of the country (*C. chordalis*, *C. hamata*, *C. jamesonii*, *C. lapazensis*, *C. pichinchensis*).

Contrarily, at least two species are found only in the dry southern half of the country, on Pacific-facing slopes. One is *C. catamarcensis*, a species from dry areas of N Chile and NW Argentina, that has its northernmost limit here in Peru and grows in association with streams. The second is the endemic *C. ros-desertum*, from desert fog oasis lomas (Muñoz-Schüler et al. in press).

### Limitations in the knowledge of the genus *Carex* in Peru

Taxonomic knowledge of *Carex* in Peru has been hindered by several issues.

A bias in botanical practice seems to play a role since *Carex*, as an herbaceous element with non-showy flowers, is often neglected during collection. Most botanists tend to favour trees and shrubs or other more apparent or showy flowering plants.

The intrinsic idiosyncrasy of the Peruvian geography also affects the lack of collections in herbaria. The studied herbaria revealed that collections seem to be less abundant in the southernmost part of the country, while the north and central regions are much better sampled (Fig. 2). It may be partly due to the lesser availability of habitats for *Carex* in the southern regions. However, the eastern limit of the cordillera is not as dry and our own work has revealed a considerable diversity, including new records. Indeed, there is a number of species reported from central and northern Peru as well as from Bolivia, but not recorded in southern Peru (*C. brachycalama*, *C. brehmeri*, *C. laegaardii*, *C. lapazensis*, *C. paramorum*). Perhaps the less developed infrastructures in the region, with fewer roads and a much rough orography, is hindering collections in these areas. This situation can be extended to several species related to Amazon-facing valleys, which are poorly collected in Peru, such as *C. polysticha*, *C. porrecta* and *C. tamana*.

Eventually, some species are diminutive components of the altiplano vegetation (e.g. *C. brachycalama*, *C. brehmeri*, *C. collumanthus*, *C. herba-alpacae*, *C. humahuacaensis*, *C. hypsipedos*, *C. melanocystis*, *C. ownbeyi*, *C. punicola*, *C. via-montana*). Some of them are acaulescent, with the inflorescences appearing immediately above the surface, often concealed by the leaves, a feature relatively frequent in Andean *Carex* compared to other regions of the World (Jiménez-Mejías et al. 2021b). Being scarcely conspicuous, some species remain unnoticed to the untrained eye, which may explain its apparent rarity.

### Preliminary conservation assessment

We are aware that information pertaining to the conservation status of *Carex* species in Peru is very limited. For example, no detailed information about population sizes or demographic tendencies is available and current and/or potential threats for any species are unknown in most cases. In addition, many species (especially those with dwarf size) have been probably under-collected, misidentified and/or overlooked, making the delimitation of its Peruvian distribution incomplete and poorly understood. This lack of information precludes the complete application of most IUCN criteria to assess if a threat category (Critically Endangered, Endangered, Vulnerable) is applicable to a species and, therefore, hinders a complete conservation assessment. Therefore, we advance that all the selected species would be formally considered as Data Defficient (DD) in Peru. Table 3 summarises the main features accounted in our preliminary conservation assessment of the selected 24 rare and/or endemic species. The preliminary evaluation placed all of them under the CR or EN categories as based only on AOO. However, several of these species may have been overlooked and our assessment could have overestimated the threat category. As a general comment, large EOOs related to few occurrences and small AOO can be interpreted as indicative of the plant being overlooked. Additional comments are provided below in the pertinent cases.

**Table 3.** Preliminary conservation assessment at national level of selected rare and/or (sub)endemic species of *Carex* from Peru, as exclusively based on Peruvian populations. Endemic and subendemic species are marked respectively with two (**) and one (*) asterisk. Number of known occurrences (number of 4 km^2^ occupied grid cells provided in brackets if different from the total number of occurrences), extent of occurrence (EOO), inferred area of occupancy (AOO) and potential IUCN evaluation under criterion B is provided. For species with occurrences reduced to 1–2 grid cells, EOO cannot be calculated.

Species	Number of occurrences	EOO (km^2^)	AOO (km^2^)	Potential IUCN category
*C. acutata*	6	24817	24	EN (B2)
*C. amicta*	4	2527	16	EN (B1+B2)
C. canoi**	24 (15)	8102	60	EN (B2)
*C. catamarcensis*	4 (3)	1510	12	EN (B1+B2)
*C. colombiana*	4 (2)	-	8	CR (B2)
C. david-smithii**	44 (36)	132430	144	EN (B2)
*C. gayana*	3 (2)	-	8	CR (B2)
*C. guaguarum*	11	4182	44	EN (B1+B2)
*C. haematopus*	2	-	8	CR (B2)
C. hebetata**	53 (38)	37413	152	EN (B2)
C. herba-alpacae*	5	2867	20	EN (B1+B2)
*C. huancabambica*	2	-	8	CR (B2)
C. hypsipedos**	3 (1)	-	4	CR (B1+B2)
*C. lepida*	1	-	4	CR (B1+B2)
*C. ownbeyi*	3	26755	12	EN (B2)
C. pachacutecii**	3	3305	12	EN (B1+B2)
*C. polysticha*	2	-	8	CR (B2)
*C. punicola*	3	8485	12	EN (B2)
*C. pygmaea*	1	-	4	CR (B1+B2)
C. regina*	19 (18)	24003	72	EN (B2)
C. roalsoniana*	3	176	12	EN (B2)
C. ros-desertum**	2	-	8	CR (B2)
*C. tamana*	1	-	4	CR (B1+B2)
*C. viamontana*	1	-	4	CR (B1+B2)

*Carex* has a higher species richness in temperate and cold areas of the Northern Hemisphere (Martín-Bravo et al. 2019). Remarkably, most *Carex* lineages which have been able to colonise South America seem to have retained a preference for relatively cold habitats. This is a pattern common in other temperate groups that entered the continent. Therefore, a large proportion of Neotropical species are confined to mountain ranges and mainland or island high latitudes. Indeed, in Peru, this is evident in the distribution of most species restricted to the Andean realms (Fig. 2). Many *Carex* species play a critical ecological role as dominant elements of some Andean herbaceous communities (e.g. grasslands like pajonales and punas, wetlands like bofedales). Given the ever increasing effects of climate change on taxa and populations at their altitudinal range edges (e.g. Breshears et al. (2008)), this problem is especially sensitive in high altitude Andean areas, where the availability of suitable habitats may be reduced, since the populations may not be able to migrate upslope to escape warming so they could face extinction as they are limited at mountain summits with no additional possibilities to ascend in altitude. In fact, most of the wet habitats where *Carex* species grows receive their water supplies from glaciers that have been calculated to be extinguished in the next few years (Polk et al. 2019) compromising the long-term survival of many populations or the species, especially for the endemic ones.

### Comments on particular species

We provide comments on certain species whose situation requires explanation in terms of their presence in Peru, their taxonomic identity and/or their conservation assessment. Names are sorted as in the systematic arrangement presented in Table 1. References to occurrences related to conservation status refer to Table 3. When a taxon is reported here for the first time for Peru, we also provide the complete information of the specimens in the main text.

#### A. Subg. *Psyllophorae* (Degl.) Peterm

##### A.1. Sect. *Junciformes* Kük.

*Carex herba-alpacae* Jim.Mejías & A.Mor.Alons.

This is a recently described species from the high Andean wetlands (“bofedales”) from Peru and Bolivia (Morales-Alonso et al. 2024a). It is included in one of the few *Carex* groups with a remarkable species richness in South America (sect. *Junciformes*, subgen. *Psyllophorae*; Benítez-Benítez et al. (2021)). It is very similar to *C. vallis-pulchrae* Phil., a species widely distributed in similar habitats across South American Patagonia and the Andes and it has been confounded with it (Wheeler and Beck 2011).

*Carex herba-alpacae* is known only from a few locations. It may have remained unnoticed in other places to date due to its diminutive size and, therefore, its distribution range is insufficiently known. In congruence, only five locations are hitherto known from a long portion of the southern half of Peruvian Andes (EOO 2867 km^2^ vs. AOO 20 km^2^), suggesting the likely existence of more populations.

*Carex setifolia* Kunze ex Kunth

This is a species currently confirmed only for Chile (Muñoz-Schüler et al. 2023; Morales-Alonso et al. 2024a). It was cited from southern Peru (Tacna) by Macbride (1936 [*Woitschach s.n.*.]). The identity of the specimen was also confirmed by Kükenthal (1909), who cited it in his world monograph of *Carex*. After a thorough search, material of this species could not be located in F (K. Hansen, pers. comm.) where Macbride was working. If the material studied by Macbride belonged to B, it was probably destroyed during WWII.

In Chile, *C. setifolia* is part of the Mediterranean-type vegetation, growing at low and medium altitudes within scrub or in rocky outcrops. Despite its presence in Tacna initially seeming improbable (Morales-Alonso et al. 2024a), the recent finding of *C. ros-desertum* associated to the desert fog oasis of southern Peru (Muñoz-Schüler et al. in press) makes the presence of *C. setifolia* in the area plausible. Further searches are required to confirm the record of this species in southern Peru.

*Carex via-montana* A.Mor.Alonso & Jim.Mejías

A Northern Andes diminutive endemic, recently segregated from *C. phalaroides* Kunth (Morales-Alonso et al. 2024a). It has only three confirmed Peruvian occurrences in two cells. Our evaluation considered it as Critically Endangered (CR). Its distribution limit in Peru may be conditioning its rarity. Although we believe that this species could be rare and considerably localised in the country, there is a possibility that it has been overlooked or simply misidentified. Further efforts are needed to confirm the status of the species in the Peruvian Andes.

#### C. Subg. *Vignea* (P.Beauv. ex T.Lestib.) Peterm.

##### C.1. Annectens clade

*Carex gayana* É.Desv.

A species with a primary Patagonian distribution, becoming scattered northwards through the Andes and marginally reaching Peru (Jiménez-Mejías et al. 2021a). We confirm that the specimen cited in Brako and Zarucchi (1993) [*Kunkel 545* SI!] belongs to this species.

*Carex gayana* may constitute a taxonomic complex pending deeper systematic studies. In contrast to southern specimens, northern collections sometimes display prickles on the utricle distal margins. In our opinion, this deserves further research to evaluate if it has taxonomic implications.

Some of the Peruvian (and Bolivian) records are based on immature specimens, which sometimes correspond to dark-coloured specimens of the closely-related *C. ecuadorica* (P.J.-M., pers. obs.). Both species can be easily distinguished when ripe by the utricle characters cited in the key. We have been able to confirm the presence of *C. gayana* in Peru through a few specimens from Puno and Junín. A thorough revision of material under *C. ecuadorica* in Peruvian herbaria would be desirable to better understand the distribution and taxonomy of *C. gayana* in the country, which constitutes the northern limit of its distribution.

In Peru, the three known occurrences correspond to only two cells. Although we consider this species could be rare and considerably localised in the country, it could have been so far overlooked or simply misidentified. Further efforts are needed to confirm the status of the species in the Peruvian Andes.

*Carex ownbeyi* G.A.Wheeler

An Andean endemic with a very wide, but poorly delimited distribution along the tropical and subtropical Andes, from North Argentina to Colombia (Jiménez-Mejías et al. 2021a). Its small size, together with the poor understanding of the taxonomy of *Carex* subg. *Vignea* by most collectors, points to the possibility that this species may have been overlooked or simply misidentified. The three hitherto known Peruvian occurrences are widely separated across the Central Andes which suggests that there could be more populations awaiting discovery.

*Carex praegracilis* W.Boott

A North American species reported from Peru by Macbride (1936). The name *C. praegracilis* has been used in the past to refer to South American species of the Annectens clade (Jiménez-Mejías et al. 2021a). We have located the specimen cited by Macbride [Junin, La Oroya, *Macbride 959*, F-517487 digital image!] and it corresponds to *C. ecuadorica*. Accordingly, the presence of *C. praegracilis* in Peru should be dismissed.

##### C.2. Sect. *Cyperoideae* G.Don

*Carex bonplandii* Kunth

The group of *C. bonplandii* is a poorly understood complex of species, widely distributed through the mountains of the Neotropics from Central America to Bolivia (POWO 2025). The group encompasses names as *C. toreadora* Steyerm., described from Ecuador (Steyermark 1964) or *C. peruvida* G.A.Wheeler (Wheeler 2002a), described from Bolivia.

Specimens that can be determined as part of the *C. bonplandii* group display a broad ecological and morphological variation, encompassing large (> 50 cm tall) plants from wet soils in open forest to dwarf (< 5 cm) specimens from high altitude bofedales. We believe that, under such variation, there is taxonomic heterogeneity. However, we refrain from presenting a taxonomic treatment at this moment until additional morphologic and/or genetic data are available, to avoid adding potential confusion to the already problematic situation.

*Carex macloviana* D’Urv

A disjunct bipolar species known in South America from Patagonia. It was cited from Cajamarca by Macbride (1936; [*Raimondi*]), who reported that the identity of the specimen was confirmed by Kükenthal. However, the collection was not cited in Kükenthal's (1909) monograph nor in its earlier revision of the genus for South America (Kükenthal 1900). The specimen has not been located in F (K. Hansen, pers. comm.) where Macbride resided. If the material studied by Macbride belonged to B, it was probably destroyed during WWII.

Another specimen [Ecuador, *Spruce 5908*] from Tropical South America initially determined by Kükenthal (1900: 506) as *C. macloviana* was later cited by himself under *C. ecuadorica* (Kükenthal, 1909: 118). This points to the fact that Kükenthal’s knowledge about Tropical Andean *Carex* was unstable at the date, probably due to the lack of accurate collections. Accordingly, we believe that the record of *C. macloviana* from Peru is erroneous and probably refers to a different species, perhaps the closely-related *C. mandoniana* or also to *C. ecuadorica*.

*Carex peucophila* Holm.

The name *C. peucophila* has been usually used to refer to specimens with light-coloured inflorescences assignable to the *C. bonplandii* group, as often found in herbarium specimens (P.J.-M., pers. obs.). Conversely, the type of *C. peucophila*, from Mexico, is much more closely related to *C. mandoniana* than to *C. bonplandii* (P.J.-M., pers. obs.). The published record *C. peucophila* Holm. from Peru (Salvador et al. 2009) actually refers to a *C. mandoniana* specimen [*Salvador et al. 793*, USM 215250!].

##### C.3. Sect. *Bracteosellae* Jim.Mejías & Muñoz-Schüler

*Carex bonariensis* Desf. ex Poir.

Until recently, *C. bonariensis* was the only species from sect. *Bracteosellae* reported from northern South America. As indicated by the epithet, it is a species described from Buenos Aires (Argentina) and primarily distributed through the temperate areas of the Southern Cone (see Jiménez-Mejías et al. (2021a)). Recent studies (Morales-Alonso et al. 2025; Muñoz-Schüler et al. in press) have revealed that the northern populations from Colombia, Ecuador and Peru are morphologically and genetically distinct from those of the Southern Cone and, in fact, belong to two different species: *C. guaguarum* and *C. ros-desertum*.

*C. guaguarum* Jim.Mejías & Muñoz-Schüler

This is the northernmost species of sect. *Bracteosellae* in South America. It is a plant from wet soils in montane forests, spanning from northern Peru to Colombia.

In Peru, it has been reported from 11 occurrences, all of them found in the Department of Cajamarca and mostly concentrated in the south part of the Department, not too far from the capital, except from one disjunct population in Lambayeque Department. None of the known populations is on protected land. All this would point to the Endangered category (EN). However, it seems that it can tolerate some degree of disturbance, with populations observed at road margins and in crop fields in Ecuador (P.J.-M. pers. obs.). Additional data are needed to confirm the endangered status of the species in Peru.

*C. ros-desertum* Jim.Mejías & Muñoz-Schüler

An exceptional, recently described Peruvian endemic species (Muñoz-Schüler et al. in press), the only *Carex* species so far reported from low altitudes in tropical latitudes. It is confined to the desert fog oasis ecosystems (Moat et al. 2021) from desert coastal areas of southern Peru, where it is known only from two populations. This is an extremely arid area in which *Carex* is almost absent, but this species inhabits coastal hills ("lomas") with maximum elevations just exceeding 1000 m a.s.l., where it seems to be able to persist thanks to the humidity these hills can obtain from the fogs coming from the sea. The two known populations were found at elevations between 600 and 800 m and are separated by ca. 400 km and correspond to an AOO = 8 km^2^, potentially pointing to the Critically Endangered category. One of the populations is located in protected land, the Private Conservation Area “Lomas de Atiquipa”, funded to protect the coastal hill forest and scrub of South Peru. The scarcity of available habitats in the regions occupied by the species in Peru, plus the anthropogenic pressure on them (Moat et al. 2021) may indicate a real global critically endangered status. Additional searches must be performed in other lomas locations in the Departments of Moquegua and Tacna.

#### D. Subg. *Uncinia* (Pers.) Peterm.

##### D.1. Sect. *Wheelerianae* Jim.Mejías, Martín-Bravo & Reznicek.

*Carex lepida* Boott

An apparently widely distributed Andean species, initially believed to be a narrow Ecuadorian endemic (Jiménez-Mejías and Escudero 2016) and subsequently found in Colombia, Bolivia (Jiménez-Mejías and Reznicek 2018) and Peru (Jiménez-Mejías et al. 2023a), as well as in additional locations in Ecuador (Jiménez-Mejías et al. 2023b). Although only a single Peruvian occurrence is known, which would place the species in the Critically Endangered (CR) category at the national level, its wide reported distribution suggests that it has likely been overlooked due to its inconspicuous morphology.

*Carex pachamamae* Jim.Mejías & Reznicek

*Carex pachamamae* is a species recently described from the yungas of Bolivia (Jiménez-Mejías and Reznicek 2018; Jiménez-Mejías et al. 2020b). Its presence in the Amazon basin-facing yungas of the southernmost areas of Peru seems possible.

*Carex roalsoniana* Jim.Mejías & M.Escudero

A recently described species endemic from the Andes of Ecuador and Peru (Jiménez-Mejías and Escudero 2016). In Peru, it is known from three occurrences in montane forest understory in Piura Department (Jiménez-Mejías et al. 2023a), of which only one is on protected land (Tabaconas Namballe National Sanctuary). The restricted range in Peru would indicate the Endangered category (EN).

##### D.2. Sect. *Uncinia* (Pers.) Baill.

*Carex brevicaulis* Thouars. [*Uncinia brevicaulis* (Thouars) Kunth]

The name *C. brevicaulis* has been widely applied to the austral *C. delacosta* Kuntze [= *Uncinia macloviana* Gaudich.] and associated species, as the tropical *C. laegaardii* J.R.Starr [= *Uncinia paludosa* G.A.Wheeler & Goetgh.]. In a strict sense, *Carex brevicaulis* is an endemic from the South Atlantic remote archipelago of Tristan da Cunha. The published report from Peru in Brako and Zarucchi (1993) [*León & Young 1663*, MO!] corresponds to *C. laegaardii*.

*Carex erinacea* Cav. [*Uncinia erinacea* (Cav.) Pers.].

The name *Uncinia longifolia* Kunth was published with the geographical indication “Peruvia nisi Chile” [Peru, except Chile]. We have located the type at G [G00098551 digital image!] and the described plant is a specimen of *C. erinacea* bearing a label indicating Peru as the collecting place. However, *C. erinacea* is mainly a Valdivian element endemic to Chile, present in the northernmost southern regions of the country, becoming rarer in the central provinces. The ecology of the species is mostly related to humid Valdivian forest (P.J.-M., S.M.-B., pers. obs.).

We consider that the report from Peru is probably a confusion. The collector of the material, J. Dombey, visited both Chile and Peru (Deschamps-Lang 1995) and possibly mislabelled it.

*Carex goetghebeurii* J.R.Starr [=*Uncinia tenuifolia* G.A.Wheeler & Goetgh.]

This is a poorly understood species only known from a single population growing in a cliff in a montane forest in the southern Ecuadorian Province of Zamora-Chinchipe (Wheeler and Goetghebeur 1995), not far from the border with Peru and which seems to have been affected by a quarry (Jiménez-Mejías et al. 2023b). It is a medium-size plant with spikes often hidden amongst the leaves. Its presence in the northernmost areas of Peru seems possible.

#### E. Subg. *Carex*

##### E.1. Decora clade

*Carex amicta* Boott

A Northern Andes endemic recently reported from Peru (Jiménez-Mejías et al. 2020a, 2023a), where it reaches its southernmost limit in the northern regions of the country, being seemingly rare and localised. Our evaluation considered it as Endangered (EN). However, it may have been overlooked given its small size. Since its distribution limit in Peru may be conditioning its rarity and given that none of the known occurrences is included in protected areas, the EN category may be taken as indicative of its conservation status in Peru, until more information is obtained.

*Carex polystachya* Sw. ex Wahlenb., *C. beckii* G.A.Wheeler and *C. sodiroi* Kük.

The name *C. polystachya* has been used as a hotchpotch to refer to a complex of species widely distributed through the Neotropical montane forest, from Mexico and the Caribbean to the north limit of the Southern Cone (POWO 2025).

The type of *C. polystachya* is from Jamaica (Wahlenberg 1803). Two names have been described from South America: *Carex beckii* from Bolivia and *C. sodiroi* from Ecuador.

Preliminary unpublished Sanger-based molecular results show that samples identified as *C. polystachya* are phylogenetically heterogeneous (Jiménez-Mejías et al. unpublished). Topotypic populations corresponding to *C. beckii* and *C. sodiroi* form two separate clades, with populations from northern Peru grouping with the first and those from southern Peru grouping with the second.

Here, we adopt a compromise solution roughly assigning populations from Cajamarca and Amazonas northwards to *C. sodiroi* and those from Pasco and southwards to *C. beckii*. We could not study material from the intermediate regions in detail, only digitised images of the vouchers being examined. These are not assigned to any taxon and listed generically as “*C. polystachya* group”, since the material could not be studied. We admit that the morphological characterisation that we have performed to distinguish between *C. beckii* and *C. sodiroi* is still vague and the identification key can probably be improved.

*Carex porrecta* Reznicek & Camelb.

This is a species described from Ecuador and known from Colombia, Venezuela and Costa Rica (Reznicek and Camelbeke 1996), here reported for the first time for Peru. The Peruvian populations seem to be highly disjunct from the closest ones from Ecuador. This may deserve further investigation to evaluate whether this remarkable disjunction is simply due to the presence of unknown intermediate populations, a relatively recent long-distance dispersal or if it has taxonomic implications.

Studied material: Cusco, Urubamba, Machu-Picchu, microcuenca Cedrobamba, 2350 m alt., matorral de hojas silicicas cortantes en lugares húmedos del bosque secundario, 02 Jan 2001, *Tupayachi et al. 4615* (CUZ-30000). Cusco, Vilcabamba, Oyara, Molinochayoc, bosque primario húmedo, 22 Feb 2007, *Valenzuela et al. 8921*, (MO sn). Cusco, muddy river bank of the Río Marcapata, 60 km above Quincemil, 1880 m alt., 17 Jan 1973, *Madison 1012*, (GH sn). Junín, Huacapistana, 1800–2400 m alt., open hillside, 6–8 Jun 1929, *Killip & Smith 24231*, (NY-02861806).

##### E.2. Sect. *Abditispicae*

*Carex ruthsatzae* G.A.Wheeler

A diminutive species known from Bolivia and N Argentina. it is reported by POWO (2025) in Peru. However we have seen no specimens of this taxon. The record in POWO may be based on a Bolivian population adjacent to the Peruvian border (Wheeler, 2002b). The presence of the species in the altiplano of Puno seems possible.

##### E.4. Hirta clade

*Carex acutata* Boott

A widely distributed tropical American endemic, reported from Bolivia to Venezuela. It seems to be a rare species in Peru, with only six known occurrences stretching across northern and central portions of the Andes, with three of them very close in Huayhuash mountain range, resulting probably in a total of four locations. However, the large EOO suggests that the species range is poorly known and there are probably many more populations in the Peruvian Andes.

*Carex haematopus* Jim.Mejías & Roalson

A recently described endemic from the Northern Andes of Colombia (Jiménez-Mejías and Roalson 2016), later reported from Ecuador (Jiménez-Mejías et al. 2018) and Peru (Jiménez-Mejías et al. 2023a). In Peru, it is only known from two relatively close occurrences in Cajamarca in northern Peru. This would qualify the species as Critically Endangered (CR) at the national level. However, some of the Cajamarca populations have been observed at the margins of roads and on grazing lands (P.J.-M. pers. obs.), so it seems that it can tolerate some degree of disturbance. Additional data are needed to confirm the endangered status of the species in Peru.

*Carex hookeri* Kunth (≡ *Carex beechyana* Boott [“beecheyana”])

*Carex hookeri* is one of the three species from the Hirta clade in South America with hairy utricles, the other three being *C. aematorrhyncha* É.Desv, *C. haematopus* and *C. tweedieana* Nees. From these, only *C. haematopus* has been reported from northern South America, being present in Peru, Ecuador and Colombia (Jiménez-Mejías and Roalson 2016; Jiménez-Mejías et al. 2023a). *Carex hookeri* is indeed a species of problematic taxonomy, described and reported from Chile and of intermediate appearance between *C. chilensis* Brong. and *C. aematorrhyncha*. (Muñoz-Schüler et al. 2023), two species only known from the Southern Cone.

We have not located the material cited from Peru by Macbride (1936 [Puno, Sachapata, *Grisebach 3290*] at F (K. Hansen, pers. comm.). The taxon was also cited from Ecuador by Kükenthal (1909). In our opinion, these records very probably correspond to *C. haematopus*.

###### Carex paramorum

Taxon classificationPlantaePoalesCyperaceae

Jim.Mejías nom. nov.

3A94FFA5-2C32-5A2A-B373-B2086B4C7800

urn:lsid:ipni.org:names:77377174-1

≡ Carex tristicha Spruce ex Boott in Ill. Gen. Carex 4: 153 [tab. 493] (1867), nom. illeg., replaced synonym, non Carex tristicha Boeckeler, Flora 41: 651 (1858).

####### Lectotype

(here designated): Ecuador, Riobamba, “in pastis montis Titaicún”, Nov 1858, *Spruce 5767* (K-000584699!; iso- K-000584697!).

**Note**. This is a species frequently confused with *C. pygmaea* (see below): It has been often treated as conspecific with the closely-related *C. tamana*, which has been partly due to the lack of a legitimate name for the taxon. *Carex paramorum* can be distinguished from *C. pygmaea* and *C. tamana* using the characters reported in the key. It is an element of mesic to wet treeless páramos, here reported for the first time for Peru, more abundant in Ecuador, Colombia and Venezuela and also known from Costa Rica (P.J.-M., pers. obs.).

Here, we coin a legitimate name for this species, based on the illegitimate *C. tristicha* Spruce ex Boott. The etymology of the epithet “paramorum” refers to the páramos where this species inhabits, meaning “of the páramo”.

####### Studied material.

Ancash, pista entre Chavín de Huantar y Catac, 9°41.2089'S, 77°14.5565'W, 4305 m alt., prados húmedos en claros de pajonal, 30 Sep 2021, *Martín-Bravo et al. 210SMB21* (UPOS sn). Cajamarca, Lagunas del Alto Perú, 6°54'32.93’'S, 78°25'45.2"W, 3938 m alt., borde de lagunas turbosas, 6 Oct 2021, *Jiménez-Mejías et al. 30PERPJM21* (UPOS sn). Cajamarca, carretera entre Chota y Cutervo, 6°27'6.3’'S, 78°45'29.98"W, 3009 m alt., claros encharcados, 7 Oct 2021, *Jiménez-Mejías et al. 40PERPJM21* (UPOS sn). Huánuco, cerro Chogopata, a 5 km de comunidad campesina Lauricocha, 4000 m alt., 10 May 2004, *Salvador et al*. 795 (USM-215251). Junín, prov. Satipo/La Convención, cordillera Vilcabamba río Ene slope, near summit of divide, 11°39'36’'S, 73°40'02"W, 3350–3400 m alt., mossy upper montane forest, 8 Jun 1997, *Boyle et al. 4218*, (F-2184452, USM sn). La Libertad, Patáz Borde con el Parque Nacional Río Abiseo, Abra Ventanas, en toda la cima, pajonal con influencia de pastoreo, 3700–3800 m alt., 23 Jul 2009, *León et al. 5456* (USM-283487). Piura, Laguna Shimbe, -5,050647, -79,463077, 3237 m, prados húmedos, *Jiménez-Mejías et al. 64PERPJM21*, UPOS sn. Piura, carretera Laguna Negra a Huancabamba, 5°05'45.96’'S, 79°30'14.14"W, 3500 m alt., pajonal, 10 Oct 2021, *Jiménez-Mejías et al. 82PERPJM21* (UPOS sn).

##### *Carex polysticha* Boeckeler

A species widely distributed in Tropical America, from Mexico and the Caribbean to northern Argentina. Its presence in Peru is only reported from two occurrences in Pasco, which would result in being listed as Critically Endangered (CR) in the country. However, its rarity may probably be due to the difficult access to its habitats on the eastern side of the Cordillera. Its presence in other areas should not be discarded.

*Carex tamana* Steyerm.

A species very closely related to *C. paramorum*, from which the distinction can sometimes be difficult. It is an element of cloud forest, appearing at lower altitudes than *C. paramorum*. Here, we report the presence of this species for the first time for Peru on the Amazon-facing slopes of the Andes, with the reported locality becoming the absolute southernmost limit of this species primarily distributed in a wide area through Colombia, Ecuador and Venezuela. As in the case of *C. polysticha*, despite our preliminary evaluation listing it as Critically Endangered (CR), its rarity could result from the difficult access to the eastern side of the Andes and it may be present in other areas.

Studied material: Cusco, bajando desde el puesto de control a la Estación Biológica Waycheqa, 13°10.9678'S, 71°35.8915 W, 3315 m alt., pajonal con macollas de *Calamagrostis* sobre suelos húmedos cubiertos de musgo, 19 Sep 2021, *Martín-Bravo et al. 122SMB21* (UPOS sn).

##### E.5. Sect. *Racemosae* G.Don

*Carex hypsipedos* C.B.Clarke

This has been a bizarre species of difficult systematic placement until the study by Morales-Alonso et al. (2024b) revealed it to be a member of sect. *Racemosae* with a highly deviant morphology. It is one of the two species of *Carex* known to have underground flowers, the other being the New Zealand *C. pilifolia* K.A.Ford (Ford 2025). It is known so far only from the mining area of La Oroya (Junín), where soils have a high content of heavy metals. It may point to this species being a metallophyte which, if confirmed, would be the first case in the genus *Carex*.

The three known Peruvian occurrences are placed within the same grid cell, which is in the vicinity of mining exploitations, potentially cataloguing it as Critically Endangered (CR). This species needs to be searched for in additional areas, as its presence in other localities may have remained unnoticed due the inconspicuousness of its flowers.

*Carex phylloscirpoides* Saldivia, S.Gebauer, Martín-Bravo & Jim.Mejías.

A diminutive plant recently described from the altiplano of Tarapacá, in northern Chile (Jiménez-Mejías et al. 2021b), associated with bofedales in mining areas, which may also point again to a possible behaviour as a metallophyte (see above). Its presence in the western altiplano of the southernmost areas of Peru seems possible. This diminutive species may easily be overlooked and confused with other small high-altitude sedges, such as species from the genera *Phylloscirpus* C.B.Clarke or *Zameioscirpus* Dhooge & Goetgh.

##### E.6. Sect. *Phacocystis* Dumort.

*Carex brehmeri* Boeckeler and *C. enneastachya* C.B.Clarke

Two closely-related taxa from sect. *Phacocystis*, the only ones from that section present in Tropical South America. The differences between the small *C. brehmeri* and the large *C. enneastachya* are poorly understood. It could be possible that some of the populations labelled as *C. brehmeri* may be mere small growing-forms of *C. enneastachya* (Jiménez-Mejías et al. 2023a, b). We follow the compromise solution of keeping the two names separately to uncover the existence of such taxonomically problematic species in Peru. Additional biosystematic data are needed to evaluate the taxonomic status of both species.

##### E.7. Sect. *Fecundae* Kük.

A group of sedges of intricate taxonomy, restricted to the Neotropics and one of the two large *Carex* sections entirely confined to Tropical latitudes (the other being the *Decora* Clade). The group displays an enormous variation that has remained hidden until now, with up to 18 out of 39 species recently described as new (Lois et al. 2025). The new described species remained unnoticed due to the lack of a comprehensive taxonomic treatment. Specimens of these new species were stored in herbaria under other names from the same section. For Peru, up to four of these newly-described species are cited in the present work: *C. canoi*, *C. colombiana*, *C. pachacutecii* and *C. regina*.

Intermediates between certain species of this group are not rare, which may point to hybridisation processes. Indeed, unpublished genomic data suggest hybridisation processes between several pairs of species and possible polyploidy (García-Moro et al. unpublished data; Lois et al. unpublished data). In Peru, we have found morphologically intermediate specimens between *C. canoi* and *C. david-smithii*, *C. david-smithii* and *C. pichinchensis*, *C. david-smithii* and *C. regina* and *C. fecunda* and *C. pichinchensis*.

*Carex canoi* Jim.Mejías & Escudero

An endemic restricted to the northern Peruvian Andes. Although it is known so far from 24 occurrences, some of them are very close to each other and included in the same grid cell, pointing to the Endangered (EN) category. Since no information is known about the potential threats for the persistence of this species population, it is currently not possible to estimate the exact number of locations. None of the species’ occurrences is located in protected land.

*Carex catamarcensis* C.B.Clarke ex Kük.

This species, the southernmost representative of sect. *Fecundae* in South America, is extremely rare and the collections are very scarce despite its large size (with flowering stems often reaching 1 m or more) and a wide distribution across Argentina, Chile (Muñoz-Schüler et al. 2023) and southern Peru (Muñoz-Schüler et al. in press). In Peru, it is known only from four occurrences located in Arequipa and Tacna, where it grows along mountain streams in otherwise arid vegetation. These records occupy just three grid cells, with a EOO of 1,510 km^2^, but an AOO of 12 km^2^, both below the threshold for the Endangered (EN) category. Indeed, two of these occurrences are found in the unprotected and heavily visited area of Cataratas de Sogay (confirmed in recent observations of iNaturalist), while another, whose current persistence is uncertain, lies near the rapidly growing city of Arequipa, likely in the Chili River gorge where a hydroelectric power project is planned. These threats could seriously compromise the long-term survival of *C. catamarcensis* populations in Peru.

*Carex colombiana* Jim.Mejías, Lois, Acedo, & A.Cano

A Northern Andes endemic with its distribution centre in Colombia (Lois et al. 2025). Only four Peruvian occurrences are known from the Northern Andes, exclusively in Cajamarca, three of them located in the same grid cell, potentially placing the species in the Critically Endangered (CR) category. Occurrences are outside protected land, although one of them is very close to the Cutervo National Park.

*Carex david-smithii* Reznicek

A Peruvian endemic widely distributed along the central and northern portions of the Andes. Although it has a relatively high number of known occurrences (44), it would still easily qualify as Endangered under the B2 criterion, due to an AOO of 144 km^2^. A few populations are reported from protected land, particularly those in the Huascaran National Park.

*Carex lemanniana* Boott

A northern South American species unknown from Peru, but reported from adjacent areas of Ecuador. It is a large and conspicuous species growing in páramos. Its presence in the northernmost areas of Peru seems possible. The previous report of *C. lemanniana* in Brako and Zarucchi (1993) [Huamacucho, *Angulo & López 1492* HUT! (miscited as “*López & Sagástegui* 1492")] corresponds to *C. regina*. Other specimens from Peru determined in herbaria as *C. lemanniana* in key actually refers to *C. canoi* (P.J.-M., pers. obs.).

*Carex pachacutecii* Jim.Mejías, Lois, García-Moro, Martín-Bravo & A.Cano

Another Peruvian endemic species exclusively known from three relatively close Andean occurrences in Cusco and Apurimac. One of them is located in the Choquequirao regional conservation area and another, very close to the boundaries of the Manu National Park. The currently known populations imply that it could be Endangered (EN).

*Carex regina* Jim.Mejías, Lois, Acedo, & A.Cano

A Peruvian subendemic, with very few documented occurrences in the neighbouring regions of Ecuador (Lois et al. 2025). A large and conspicuous species with one of the largest inflorescences of *Carex*, a densely-flowered many-branched panicle (Fig. 1). Our evaluation listed it as Endangered (EN).

##### E.8. Castanea clade

*Carex hebetata* Boott

A Peruvian endemic, known from a relatively high number of occurrences (53) across the northern-central part of the country. Although it is the species with the highest number of known occurrences amongst those selected here for our approach to its conservation status, it could be potentially considered as Endangered due to its restricted AOO (152 km^2^), in a similar case to that of *C. david-smithii*. In fact, a considerable number of occurrences are included in the same 4 km^2^ grid cell, reducing the number of locations. This is clearly reflecting the need for a better understanding of the conservation status of Peruvian *Carex*.

*Carex huancabambica* Gonz.Gallego & Jim.Mejías

A recently-described species from Ecuador and Peru, similar to *C. boliviensis*, only known from two occurrences from the northernmost part of the country (González-Gallego et al. 2025). Due to the small size of this species and somehow difficult distinction from the related *C. boliviensis*, it may have easily been overlooked. However, its distribution limit in Peru may also be conditioning its rarity here. Potentially classifiable as Critically Endangered (CR) due to a restricted AOO (8 km^2^).

*Carex pygmaea* Boeckeler

Previously reported from Peru (Huánuco) by Salvador et al. (2009). The cited specimen [Huánuco, Lauricocha, Salvador et al. 795, USM!] actually belongs to *C. paramorum*. However, our own fieldwork confirmed the presence of the species in Peru, but in the Department of Piura (Jiménez-Mejías et al. 2023a). As in the case of *C. huancabambica*, its distribution limit in Peru may be conditioning its rarity and that the only known occurrence lies in non-protected areas subject to heavy pressure by touristic activity (Laguna de las Huaringas). The EN category may be taken as indicative of its conservation status in Peru until more information is obtained.

Studied material: Piura, camino a la Laguna Negra, -5,061101, -79,488200, 3553 m alt., prados húmedos, 10 Oct 2021, *Jiménez-Mejías 81PERPJM21* (UPOS sn).

##### E.10. Sect. *Acrocystis* Dumort.

*Carex punicola* D.B.Poind., Jim.Mejías & M.Escudero

Another Andean endemic with a very wide, but poorly delimited distribution along the Andes, from Argentina to Ecuador (Poindexter et al. 2017; Jiménez-Mejías et al. 2023b). Its taxonomic delimitation and biogeographic knowledge have long remained poorly known (Poindexter et al. 2017), due to its dwarf size, coupled with extreme reduction and homoplastic patterns of diagnostic morphological features. Therefore, the three confirmed occurrences of this species in Peru are likely largely misrepresenting the Peruvian distribution of this species.

### Identification Key – Clave de Identificación

Five species not known in Peru, but known from adjacent territories of neighbouring countries (Bolivia, Chile and Ecuador), are included in the key, since their occurrence in Peru seems possible. These species are denoted with an asterisk (*).

By rachilla, we refer to a bristle-like organ that is contained within the utricle together with the nutlet. In the species formerly placed in the genus *Uncinia* the rachilla protrudes from the utricle beak and ends in a conspicuous hook. In other species, the rachilla may be entirely contained within the utricle or be shortly-protuding and linear.

#### Key in English

**Table:** 

1	Inflorescence a single androgynous spike at the end of the stem	**2**
–	Inflorescence with > 1 spike	**12**
2	Utricles with an exserted filament-like rigid rachilla protruding from the beak, the rachilla ending in a hook	**3**
–	Utricles without exserted rachilla, the rachilla absent or contained within the utricle or, if rachilla protruding, then it does not form a hook at its tip	**7**
3	Anther filaments filiform, narrower than the anthers; stems usually < 15 cm long, sometimes acaulescent	***C. meridensis***
–	Anther filaments flat at least proximally, the entire filament or the flat portion as wide as or wider than the anthers; stems usually > 20 cm long	**4**
4	Rachillas from the middle portion of the spike strongly spreading (the main rachilla axis form a > 45° angle with the spike axis), the hook conspicuously curved (the hook forms a > 45° angle with the proximal part of the rachilla)	***C. hamata***
–	Rachillas from the middle portion of the spike ascending (the main rachilla axis form a < 45° angle with the spike axis), the hook not or little curved	**5**
5	Spikes > 4 mm wide at its widest point (excluding rachillas), conspicuously wider distally than proximally	***C. koyamae***
–	Spikes < 4 mm wide (excluding rachillas), the width constant along the spike’s entire length	**6**
6	Leaves 0.5–1.5 mm wide, weakly herbaceous; utricles 2.5–3.3 mm	C. goetghebeurii*
–	Leaves 3–5.5 mm wide, subcoriaceous; utricles 4–5.4 mm	***C. laegaardii***
7	Utricles with a rachilla protruding from the beak; spikes with the utricles deflexed when ripe; stigmas 3	**8**
–	Utricles without rachilla or with the rachilla contained within, not protruding from the beak; spikes with the utricles ascending; stigmas 2–3	**9**
8	Pistillate glumes shorter than the utricles, brownish; staminate flowers 5–6 per spike; utricles 3–12 per spike, short stipitate, when deflexed appearing subsessile on the spike rachis; rachilla shallowly longitudinally grooved	***C. microglochin***
–	Pistillate glumes as long as or longer than the utricles, reddish-brown; staminate flowers 2–3 per spike; utricles (2)3–4(5) per spike, long-stipitate; when deflexed, the stipe appearing inverted U-shaped; rachilla conspicuously 2–3-nerved	***C. camptoglochin***
9	Plants acaulescent, the spike sessile and concealed by the leaves; stigmas 3, more or less sinuose; utricles 2.4 × 1.2 mm	C. phylloscirpoides*
–	Plants with stems, the spike exserted above the ground surface; stigmas 2–3, sinuose or curled; utricles 2.5–5 *×* 1.2–1.6 mm	**10**
10	Utricles sparsely pubescent, at least distally	C. setifolia*
–	Utricles glabrous	**11**
11	Utricles 4.2–5 × 1.3–1.5 mm, narrowly elliptical; stigmas more or less sinuose	***C. herba-alpacae***
–	Utricles 2.5–3.1 × 1.2–1.6 mm, broadly elliptical; stigmas conspicuously curled backwards	***C. lepida* p.p**.
12	At least the terminal spike gynaecandrous (with staminate flowers proximally and pistillate flowers distally; very rarely tipped by a few staminate flowers)	**13**
–	Spikes unisexual or androgynous (with pistillate flowers proximally and staminate flowers distally)	**21**
13	All spikes gynaecandrous, sessile, forming a spicate inflorescence	**14**
–	Terminal spike gynaecandrous, at least some lateral spikes entirely pistillate (rarely androgynous), the spikes sessile or with well-developed peduncles, arranged in racemose inflorescences	**15**
14	Utricle adaxial side conspicuously pluri-nerved to its middle, rarely nerveless; utricle outline narrowly ovate, narrowly elliptic or fusiform; stems straight or curved	***C. bonplandii***
–	Utricle adaxial side nerveless or very weakly nerved at its base, rarely 1–2 lateral nerves reaching its middle; utricle outline broadly ovate to broadly elliptic; mature stems curved	***C. mandoniana***
15	Utricles glabrous, 3–6 mm long, constricted at apex into a ± bifid beak; leaves glabrous; plants robust, with stems usually > 20 cm long, straight, not flexuose	**16**
–	Utricles glabrous, pilose or pubescent, < 3.6 mm long, constricted at apex into a truncate or bidentate beak or beakless; plant with conspicuous stems or acaulescent, the stems straight or flexuose	**17**
16	Glumes hyaline, straw-coloured, pale-brown or orangish; utricles 4–6 mm long	***C. polysticha p.p***.
–	Glumes dark reddish-brown to purplish; utricles 3–4 mm long	***C. acutata p.p***.
17	Utricles densely pubescent, tipped by a conspicuous bifid beak, the beak teeth divergent, separated by a U-shaped sinus; glumes purplish; leaves glabrous	***C. haematopus* p.p**.
–	Utricles glabrous or sparsely pilose, beakless or tipped by a truncate, bidenate or bifid beak, the beak teeth then ± parallel and separated by a V-shaped sinus; glumes hyaline, straw-coloured, pale-brown, orangish or reddish-brown; leaf surface glabrous or pilose	**18**
18	Utricles constricted at apex into a conspicuous truncate or bidendate beak 0.4–0.7 mm long	**19**
–	Utricles beakless or nearly so, attenuated into a cuneate tip	**20**
19	Plants acaulescent or nearly so; leaves glabrous	***C. punicola* p.p**.
–	Plants with well-developed stems; leaves pilose	***C. crinalis* p.p**.
20	Stems at maturity elongated and flexuose, usually prostrate or decumbent, several times longer than the leaves; utricles narrowly elliptical to ovate	***C. boliviensis* ssp. boliviensis**
–	Stems at maturity straight, erect, < 2× as long as the leaves, sometimes acaulescent plants; utricles broadly elliptical to elliptical-obovate	***C. huancabambica***
21	Leaves pubescent or ciliate -with soft hairs- at least proximally	**22**
–	Leaves glabrous or with scabrid projections on the surface	**23**
22	Leaves 3–5.5 mm wide, subcoriaceous, ciliate on the margins and abaxial middle nerve, the surfaces glabrous; inflorescence often acaulescent, the stem very short, concealed and much surpassed by the leaves	***C. pygmaea***
–	Leaves 1.5–4 mm wide, weak to ± rigid, with the margins, nerves and the abaxial surface pubescent at least proximally, rarely the abaxial surface entirely glabrous; stems usually well-developed, longer than or equalling the leaves	***C. crinalis* p.p**.
23	Utricles with faces pubescent or pilose	**24**
23	’ Utricles entirely glabrous except sometimes the beak, or all flowers staminate	**29**
24	Inflorescence much branched, paniculate, with utriculiform cladoprophylls at the base of some of the branches and spikes (an utricle-like structure from whose apex the inflorescence branch protrudes)	***C. amicta***
–	Inflorescence racemose or spicate, the lateral spikes not branched; cladoprophylls at the base of the inflorescence lateral branches tubular, scale-like or not apparent	**25**
25	All spikes androgynous, the staminate portion usually concealed by the utricles; glume sides hyaline; utricle beak truncate or mamellate	**26**
–	Proximal spikes pistillate, very rarely all the spikes androgynous, the staminate portion usually apparent, well-exserted above the utricles; glume sides, blackish, purplish, reddish-brown, brown, orangish or straw-coloured, rarely hyaline; utricle beak bifid or bidentate	**27**
26	Plant acaulescent or fertile culms ≤ 2 cm; leaves ≤ 7 cm long; utricles laxly pubescent, with the hairs associated with the nerves	***C. via-montana***
–	Plants with well-developed culms, usually > 5 cm, rarely acaulescent; leaves usually > 10 cm long; utricles densely pubescent, the hairs ± regularly distributed all over the utricle body	***C. melliza***
27	Utricle beak ciliate, bidentate at apex; glumes dark, blackish, purplish or dark reddish-brown	***C. canoi* p.p**.
–	Utricle beak scabrid or smooth, bidentate or bifid at apex; glumes dark to pale, purplish, reddish-brown, brown, orangish or straw-coloured, rarely hyaline	**28**
28	Utricles densely pubescent, tipped by a conspicuous bifid beak, the beak teeth divergent, separated by a U-shaped sinus; glumes purplish	***C. haematopus* p.p**.
–	Utricles glabrous or sparsely pilose, by a truncate or bifid beak, the beak teeth ± parallel and separated by a V-shaped sinus; glumes hyaline, straw-coloured, pale-brown, orangish or reddish-brown	***C. punicola* p.p**.
29	Cladoprophylls utriculiform present (at the base of some inflorescence branches and spikes, there is an utricle-like structure from whose apex the inflorescence branch protrudes)	**30**
–	Cladoprophylls tubular, funnel-form, scale-like or not apparent	**33**
30	Inflorescence open, paniculiform; utricles without rachilla [a linear scale or bristle-like structure contained within the utricle together with the nutlet]	**31**
–	Inflorescence ± congested, subcapitate; utricles with a linear rachilla inside, protruding or not from the beak	**7 [abnormal inflorescences in unispicate species]**
31	Utricle beak conspicuosly bent, as long as or longer than the utricle body	***C. porrecta***
–	Utricle beak straight or slightly bent, shorter than utricle body	**32**
32	Glumes brown to purple-brown, contrasting with the subtending utricles; utricles 1.5–3 mm; plants from the northern Departments of Peru	***C. sodiroi***
–	Glumes pale-brown, straw-coloured or hyaline, not contrasting with the subtending utricles; utricles 2–3.5 mm; plants from the central and southern departments of Peru	***C. beckii***
33	Distal-most spike (s) entirely staminate and lateral ones entirely pistillate or androgynous (with pistillate flowers proximally and a few staminate flowers distally) or distal-most spike androgynous and lateral ones pistillate	**34**
–	Spikes ± similar in appearance, all androgynous (with pistillate flowers proximally and a few staminate flowers distally) or inflorescence with all spikes unisexual	**46**
34	Stigmas 2	**35**
–	Stigmas 3	**40**
35	Spikes cylindrical, easily told apart one from each other, giving to the inflorescence a racemiform appearance; glumes with apex blunt, purplish-black to dark brown; stems well developed or plants acaulescent	**36**
–	Spikes shortly oblong, overlapping one with another and often not easily told apart, giving to the inflorescence a spicate or subcapitate appearance; glumes with apex blunt, acute or mucronate, pale reddish-brown, straw-coloured or hyaline; plants acaulescent, the spikes often concealed by the leaves	**37**
36	Plants with short stems, < 15 cm long, often acaulescent; at least some glumes from the middle part of the spike with a conspicuous pale mid-vein 0.2–0.4 mm wide; utricles with the nerves conspicuously surpassing utricle half-length	***C. brehmeri***
–	Plants with well-developed stems, usually > 20 cm long; glumes from the middle part of the spike with a narrow pale mid-vein 0.1–0.2 mm wide; utricles nerveless or faintly nerved, the nerves usually disappearing before reaching utricle half-length	***C. enneastachya***
37	At least some spikes pedunculate	***C. hypsipedos* p.p**.
–	All spikes sessile, concealed by leaves	**38**
38	Utricles oblong, 4–8 mm long, yellowish-green to yellowish-brown or yellow; leaves less than 1.5(2) cm long	***C. collumanthus***
–	Utricles ovate to elliptic, 3.5–4.6 mm long, brownish or reddish-brown; leaves 0.5–4 cm long	**39**
39	Staminate glumes reddish-brown, tips obtuse; leaves 1.4 mm wide or less	*C. ruthsatzae* * p.p.
–	Staminate glumes straw-coloured, lanceolate, tips acuminate; leaves 1.2–3.5 mm wide	***C. humahuacaensis***
40	Utricles papillose; leaves conspicuously bluish-green	**41**
–	Utricle surface smooth; leaves green	**42**
41	Stems usually > 15 cm long; utricles fusiform; leaves channelled	***C. livida***
–	Plants acaulescent, if stems developed, then < 15 cm long; ripe utricles inflated; leaves flat	***C. brachycalama***
–	Leaves < 2.5 mm wide; utricles 2.4–3.3, constricted at apex into a truncate or bidentate beak; plant acaulescent or nearly so	***C. punicola* p.p**.
42	Leaves usually > 3 mm wide; utricles 3–6 mm long, constricted at apex into a ± bifid beak; plants acaulescent or with well-developed stems	**43**
43	Spikes ovoid to shortly oblong, usually each with < 20 utricles; plants acaulescent or with well-developed stems	**44**
–	Spikes cylindrical, usually each with > 50 utricles; plants with well-developed stems, usually > 30 cm long	**45**
44	Leaves coriaceous, the middle portion of the adaxial side smooth, irregularly wart-muricate and/or very minutely and sparsely scabrid; plants acaulescent or nearly so, the inflorescences subsessile, much shorter than the leaves, often concealed by them	***C. paramorum***
–	Leaves herbaceous to subcoriaceous, the middle portion of the adaxial side conspicuously scabrid; plants with well-developed stems, the inflorescences shorter, equalling or longer than the leaves	***C. tamana***
45	Glumes hyaline, straw-coloured, pale-brown or orangish; utricles 4–6 mm long	***C. polysticha* p.p**.
–	Glumes dark reddish-brown to purplish; utricles 3–4 mm long	***C. acutata* p.p**.
46	All spikes sessile or subsessile, separated by short internodes and usually overlapping, giving to the inflorescence a dense spicate appearance; glumes muticous or with the mid-nerve continued into an exserted mucro < 0.6 mm long	**47**
–	Spikes and/or inflorescence with at least some well-developed peduncles [difficult to observe in acaulescent plants] and often visible internodes, the last-order spikes overlapping or not, the whole inflorescence appearing spicate, racemose or paniculate; glumes muticous, mucronate or with an awn up to 1.6 mm long	**59**
47	Flowering stems shortly developed, the inflorescence sessile or even buried amongst leaves	**48**
–	Flowering stems more or less well developed, the inflorescence exserted above the leaves	**50**
48	Leaf blades flat, 1.2–4.5 mm wide; glumes straw-coloured	***C. humahuacaensis* p.p**.
–	Leaf blades filiform or incurved, 0.5–1.5(2.5) mm wide; most utricles longer than the glumes, the glumes brown to reddish-brown; inflorescence globose, pyramidal or broadly ovate	**49**
49	Inflorescence bractless or with glumaceous bracts embracing the lowermost spikes, but not the entire inflorescence; utricles nerveless or faintly nerved at the base	***C. melanocystis* p.p**.
–	Inflorescence with a setaceous bract embracing its base, usually longer than the inflorescence; utricles conspicuously nerved	*C. ruthsatzae* * p.p.
50	Inflorescence paniculate, with at least some well-developed lateral branches	**51**
–	Inflorescence capitate or spike-like, congested, without evident lateral braches	**53**
51	Glumes without hyaline margins or with very narrow ones	***C. david-smithii* p.p**.
–	Glumes with well-developed hyaline margins, wider than 0.2 mm at least on its distal part	**52**
52	Pistillate glumes reddish to purplish-red, ovate to obovate, 1.5–1.9 mm wide; utricles pale–coloured, usually profusely red–spotted, achenes invaginated or asymmetrical	***C. catamarcensis* p.p**.
–	Pistillate glumes purplish-black, lanceolate, 1.0–1.4 mm wide; utricles blackish on the upper half, not spotted or with few blackish spots towards the base; achenes symmetrical.	***C. pachacutecii* p.p**.
53	All flowers staminate or pistillate	***C. gayana* p.p**.
–	Flowers staminate and pistillate within the same inflorescence	**54**
54	Utricles verrucose or wrinkled adaxially	**55**
–	Utricles adaxially smooth, not verrucose nor wrinkled	**56**
55	Beak usually shorter than the half of the utricle body length	***C. guaguarum***
–	Beak subequal to longer than the utricle body length	***C. ros-desertum***
56	Utricles ≤ 3 mm long, markedly ovate, widest at base or immediately above it, nerveless or abaxially nerved in its lower half; beak < 1.2 mm, long incised abaxially, the incision often extending into the utricle body	**12. *C. gayana* p.p**.
–	Without the above combination of characters	**57**
57	Adaxial face of utricles with (3)4–10 raised nerves running most of the length of the utricle body	***C. ownbeyi***
–	Adaxial face of utricles nerveless or with up to 3 faint nerves	**58**
58	Most utricles longer than the glumes, the glumes dark brown; leaf blades filiform, 0.5–1.5(2.5) mm wide; inflorescence globose, pyramidal or broadly ovate, often shorter than the leaf blades	***C. melanocystis***
–	Most utricles as long as or shorter than glumes, the glumes hyaline to light brown; leaf blades filiform to flat, usually > 1.5 mm wide; and inflorescence globose, elliptical or oblong, usually surpassing the leaf blades	***C. ecuadorica***
59	Glumes blackish, purplish or dark reddish-brown, rarely hyaline; plants with flowering stems well-developed; inflorescences paniculate to racemose - if glumes hyaline, then inflorescence paniculate	**60**
–	Glumes hyaline, greenish, straw-coloured or pale reddish-brown; plants with flowering stems well-developed to acaulescent; inflorescence racemose to spicate	**72**
60	Flowers with 2 stigmas	**61**
–	Flowers with 3 stigmas	**69**
61	1^st^ or 2^nd^ order lateral inflorescence branches with the distal portion much compacted, bearing many small, crowded ovoid to shortly-oblong spikes that are difficult to tell apart	**62**
–	Lateral inflorescence branches with the terminal portion bearing ovate, oblong or cylindrical spikes that are easy to tell apart	**65**
62	Utricles attenuated into a beak ca. 1 mm long, the utricle upper third scabrid; middle and lower lateral branches of the inflorescence rather lax proximally, its axis usually visible between the spikes up to its distal third	***C. regina***
–	Utricle attenuated or constricted into a beak < 1 mm long, the utricle typically smooth, rarely with a few prickles on the beak; middle lateral branches of the inflorescence dense, its axis usually not visible between the spikes or only at its proximal half	**63**
63	Glumes without hyaline margins or with very narrow ones	***C. david-smithii* p.p**.
–	Glumes with well-developed hyaline margins, wider than 0.2 mm at least on its upper part	**64**
64	Pistillate glumes reddish to purplish-red, rarely hyaline, ovate to obovate, 1.5–1.9 mm wide; utricles pale–coloured, usually profusely red–spotted, achenes invaginated or asymmetrical	***C. catamarcensis* p.p**.
–	Pistillate glumes purplish, lanceolate, 1.0–1.4 mm wide; utricles blackish on the upper half, not spotted or with few blackish spots towards the base; achenes symmetrical.	***C. pachacutecii* p.p**.
65	Utricle constricted at top into a parallel-sided beak conspicuously bifid, usually the upper margins setulose, rarely all the utricles entirely smooth; utricles conspicuously nerved	*C. lemanniana* *
–	Utricle more or less attenuated into the beak, truncate or very shortly bidentate, the upper margins smooth or ciliolate, rarely sparsely setulose; utricles nerved or nerveless	**66**
66	Utricle margins densely ciliolate on its upper third	***C. canoi* p.p**.
–	Utricle margins smooth or, rarely, sparsely setulose	**67**
67	Inflorescence racemose or, if ± paniculate, the proximal branches arising single from each node and branching only once; utricles elliptic to elliptic-obovate	***C. pichinchensis***
–	Inflorescence an open panicle with the proximal branches one–several times ramified and bearing numerous spikes and/or several peduncles arising from a single node; utricles elliptic, suborbicular or obovate	**68**
68	Pistillate glumes reddish–brown, 2.5–3.9 × 0.7–1.2 mm; utricle beak truncate	***C. fecunda***
–	Pistillate glumes black, 4.0–5.3 × 0.7–1.6 mm; utricle beak shortly bidentate	***C. perprava***
69	Proximal-most bract conspicuously sheathing	***C. hebetata***
–	Proximal-most bract sheathless	**70**
70	Most glumes from the middle part of the main spikes with the membranaceous body not reaching the utricles tip	***C. chordalis***
–	Glumes from the middle part of the main spikes with the membranaceous body reaching or surpassing the utricles tip	**71**
71	Utricles narrowly ovate, narrowly elliptic or oblong, 3–4 × 0.9–1.3(1.5) mm, with raised nerves, ± attenuated into the beak; nutlets narrowly elliptic to oblong	***C. jamesonii***
–	Utricles obovate to suborbicular, 2.3–3.2 × 1.2–1.5 mm, nerveless or with raised nerves; nutlets markedly obovate	***C. colombiana***
72	Stigmas 2	**73**
–	Stigmas 3	**74**
73	Basal sheaths pale brown to straw-coloured; stigmas ± sinuose; stems up to 3 cm tall; underground spikes sometimes present, completely concealed by the basal sheaths of the shoots	***C. hypsipedos* p.p**.
–	Basal sheaths reddish-brown to purplish; stigmas conspicuously curled backwards; stems usually > 3 cm tall; all underground spikes absent	***C. lepida* p.p**.
74	At least proximal-most spikes pending; stigmas ± sinuose	***C. lapazensis***
–	All spikes erect; glumes muticous; stigmas conspicuously curled backwards	**75**
75	Utricles 2.7–3.3 mm long, with a beak less than 0.3 mm long or beakless; widest leaves 5–8 mm wide, subcoriaceous, bluish-green	*C. pachamamae* *
–	Utricles 3.6–4 mm long, with a beak 0.6–1.2 mm long; widest leaves up to 3.5–4 mm wide, herbaceous, green	***C. roalsoniana***

#### Clave en español

**Table:** 

1	Inflorescencia formada por una única espiga andrógina en el extremo del tallo	**2**
–	Inflorescencia con >1 espiga	**12**
2	Utrículos con una raquilla rígida que sobresale del pico, la raquilla con la forma de un filamento que termina en un gancho	**3**
–	Utrículos sin raquilla exserta, ya sea porque la raquila esté ausente o contenida dentro del utrículo, si la raquila sobresale del pico del utrículo, entonces no forma un gancho en su extremo	**7**
3	Filamentos de las anteras filiformes, más estrechos que las anteras; tallos generalmente de <15 cm de longitud, a veces plantas acaulescentes	***C. meridensis***
–	Filamentos de anteras planos al menos en su porción proximal, todo el filamento o la porción plana tan ancha o más que las anteras; tallos generalmente de >20 cm de longitud	**4**
4	Raquillas de la porción media de la espiga patentes (el eje principal de la raquilla forma un ángulo >45° con el eje de la espiga), el gancho visiblemente curvado (el gancho forma un ángulo >45° con la parte proximal de la raquilla)	***C. hamata***
–	Raquillas de la porción media de la espiga ascendentes (el eje principal de la raquilla forma un ángulo <45° con el eje de la espiga), el gancho poco o nada curvado respecto al eje de la raquilla	**5**
5	Espigas (excluidas las raquillas) >4 mm en su punto más ancho, cada espiga notablemente más ancha en su porción más distal que el tramo proximal	***C. koyamae***
–	Espigas de <4 mm de ancho (excluidas las raquillas), el ancho de la espiga constante a lo largo de toda su longitud	**6**
6	Hojas de 0,5–1,5 mm de ancho, de textura herbácea; utrículos 2,5–3,3 mm	*C. goetghebeurii* *
–	Hojas de 3–5,5 mm de ancho, subcoriáceas; utrículos de 4–5,4 mm	***C. laegaardii***
7	Utrículos con una raquilla linear que sobresale del pico; espigas con los utrículos reflejos cuando maduran; estigmas 3	**8**
–	Utrículos sin raquilla o con la raquilla contenida en su interior, sin sobresalir por el pico; espigas con los utrículos ascendentes; estigmas 2–3	**9**
8	Glumas pistiladas más cortas que los utrículos, de color marrón; flores estaminadas 5–6 por espiga; utrículos 3–12 por espiga, cortamente estipitados, cuando maduran y se hacen reflejos parecen subsésiles en el raquis de la espiga; raquilla superficialmente estriada longitudinalmente	***C. microglochin***
–	Glumas pistiladas tan o más largas que los utrículos, de color marrón rojizo; flores estaminadas 2–3 por espiga; utrículos (2)3–4(5), largamente estipitados, cuando maduran y se hacen reflejos el estípite adquiere forma de U invertida; raquilla con 2–3-nervios visibles	***C. camptoglochin***
9	Plantas acaulescentes, la espiga sésil, más o menos oculta por las hojas; estigmas 3, más o menos sinuosos; utrículos 2,4 × 1,2 mm	*C. phylloscirpoides* *
–	Plantas con tallos, la espiga sobresale de la superficie del suelo; estigmas 2–3, sinuosos o rizados; utrículos 2,5–5 *×* 1,2–1,6 mm	**10**
10	Utrículos pilosos, at menos distalmente	*C. setifolia* *
10	Utrículos glabros	**11**
11	Utrículos 4,2–5 × 1,3–1,5 mm, estrechamente elípticos; estigmas más o menos sinuosos	***C. herba-alpacae***
–	Utrículos 2,5–3,1 × 1,2–1,6 mm, anchamente elípticos; estigmas visiblemente rizados hacia atrás	***C. lepida* p.p**.
12	Al menos la espiga terminal ginecandra (con flores masculinas proximales y flores femeninas distales; muy raramente rematada por unas pocas flores estaminadas)	**13**
–	Espigas unisexuales o andróginas (con flores pistiladas proximales y flores estaminadas distales)	**21**
13	Todas las espigas ginecandras, sésiles, formando una inflorescencia espiciforme	**14**
–	Espiga terminal ginecandra, al menos algunas espigas laterales enteramente pistiladas (raramente andróginas), las espigas sésiles o con pedúnculos bien desarrollados, dispuestas en inflorescencias racemosas	**15**
14	Cara adaxial del utrículo conspicuamente plurinervada desde la base hasta su mitad, rara vez sin nervios; contorno del utrículo estrechamente ovado, estrechamente elíptico o fusiforme; tallos rectos o curvados	***C. bonplandii***
–	Cara adaxial del utrículo sin nervios o con nervios muy débilmente marcados únicamente en su base, rara vez con 1 o 2 nervios laterales que alcanzan la mitad del utrículo; contorno del utrículo de ovado a elíptico; tallos maduros curvados	***C. mandoniana***
15	Utrículos glabros, de 3–6 mm de longitud, constreñidos en el ápice en un pico ± bífido; hojas glabras; plantas robustas, con tallos generalmente de >20 cm de longitud, rectos, no flexuosos	**16**
–	Utrículos glabros, pilosos o pubescentes, <3,6 mm de longitud, constreñidos en el ápice en un pico truncado o bidentado, o sin pico; planta con tallos conspicuos o acaulescentes, los tallos rectos o flexuosos	**17**
16	Glumas hialinas, de color pajizo, marrón pálido o anaranjado; utrículos de 4–6 mm de longitud	***C. polysticha* p.p**.
–	Glumas de color marrón rojizo oscuro a purpúreo; utrículos de 3–4 mm de longitud	***C. acutata* p.p**.
17	Utrículos densamente pubescentes, rematados en un pico conspicuamente bífido, los dientes del pico divergentes, separados por un seno en forma de U; glumas purpúreas; hojas glabras	***C. haematopus* p.p**.
–	Utrículos glabros o laxamente pilosos, sin pico o rematados en un pico truncado, o si bidentado o bífido los dientes del pico entonces ± paralelos y separados por un seno en forma de V; glumas hialinas, de color pajizo, marrón pálido, anaranjado o marrón rojizo; superficie de las hojas glabra o pilosa	**18**
18	Utrículos constreñidos en el ápice en un pico conspicuo de 0,4–0,7 mm de longitud, truncado o bidentado	**19**
–	Utrículos sin pico o casi, con el ápice atenuado en una punta cuneiforme	**20**
19	Plantas acaulescentes o casi; hojas glabras	***C. punicola* p.p**.
–	Plantas con tallos bien desarrollados; hojas pilosas	***C. crinalis* p.p**.
20	Tallos alargados y flexuosos en la madurez, generalmente postrados o decumbentes, varias veces más largos que las hojas; utrículos estrechamente elípticos a ovados	***C. boliviensis* ssp. boliviensis**
–	Tallos en la madurez, rectos, erectos, <2× tan largos como las hojas, a veces plantas acaulescentes; utrículos anchamente elípticos a elíptico-obovados	***C. huancabambica***
21	Hojas pubescentes o ciliadas –con pelos suaves– al menos proximalmente	**22**
–	Hojas glabras o con proyecciones escábridas en la superficie	**23**
22	Hojas de 3–5,5 mm de ancho, subcoriáceas, ciliadas en los márgenes y el nervio medio en la cara abaxial, las superficies glabras; inflorescencia a menudo acaulescente, el tallo muy corto, oculto y muy sobrepasado por las hojas	***C. pygmaea***
–	Hojas de 1,5–4 mm de ancho, de textura herbácea a ± rígidas, con márgenes, nervios y la superficie abaxial pubescentes, al menos en la porción proximal; rara vez la superficie abaxial completamente glabra; tallos generalmente bien desarrollados, más largos o iguales a las hojas	***C. crinalis* p.p**.
23	Utrículos con caras pubescentes o pilosas	**24**
23	’ Utrículos enteramente glabros, excepto a veces el pico, o inflorescencias con todas las flores estaminadas	**29**
24	Inflorescencia muy ramificada, paniculada, con cladoprofilos utriculiformes en la base de algunas de las ramas y espigas (una estructura parecida a un utrículo anchamente abierto de cuyo ápice sobresale la rama de la inflorescencia)	***C. amicta***
–	Inflorescencia racemosa o espiciforme, las espigas laterales no ramificadas; cladoprofilos en la base de las ramas laterales de la inflorescencia tubulares, escamosos o no aparentes	**25**
25	Todas las espigas andróginas, la porción estaminada generalmente oculta por los utrículos; glumas con los lados hialinos; pico del utrículo truncado o mamelado	**26**
–	Espigas proximales pistiladas, muy raramente todas las espigas andróginas, la porción estaminada usualmente aparente, exserta por encima de los utrículos; glumas con los lados negruzcos, purpúreos, marrón rojizos, marrones, anaranjados o pajizos, raramente hialinos; pico del utrículo bífido o bidentado	**27**
26	Planta acaulescente o con tallos fértiles ≤ 2 cm; hojas ≤ 7 cm de longitud; utrículos laxamente pubescentes, con los pelos asociados a los nervios	***C. via-montana***
–	Plantas con tallos bien desarrollados, generalmente de más de 5 cm, rara vez acaulescente; hojas de más de 10 cm de longitud; utrículos densamente pubescentes, con pelos distribuidos regularmente por todo el cuerpo del utrículo	***C. melliza***
27	Pico utrículo ciliado, de ápice bidentado; glumas oscuras, negruzcas, purpúreo o marrón rojizas oscuras	***C. canoi* p.p**.
–	Pico utrículo escábrido o liso, de ápice bidentado o bífido; glumas oscuras a pálidas, purpúreas, marrón rojizas, marrones, anaranjadas o pajizas, raramente hialinas	**28**
28	Utrículos densamente pubescentes, rematados en un pico conspicuamente bífido, los dientes del pico divergentes, separados por un seno en forma de U; glumas purpúreas	***C. haematopus* p.p**.
–	Utrículos glabros o laxamente pilosos, rematados en un pico truncado, o si bidentado o bífido los dientes del pico entonces ± paralelos y separados por un seno en forma de V; glumas hialinas, de color pajizo, marrón pálido, anaranjado o marrón rojizo	***C. punicola* p.p**.
29	Cladoprofilos utriculiformes presentes (en la base de algunas ramas y espigas de la inflorescencia hay una estructura parecida a un utrículo anchamente abierto de cuyo ápice sobresale la rama de la inflorescencia)	**30**
–	Cladoprofilos en la base de las ramas laterales de la inflorescencia tubulares, en forma de embudo, escuamiformes o no aparentes	**33**
30	Inflorescencia abierta, paniculiforme; utrículos sin raquilla [una escama lineal o estructura similar a una cerda contenida dentro del utrículo junto con la nuececilla]	**31**
–	Inflorescencia ± congestionada, subcapitada; utrículos con una raquilla lineal en su interior, que sobresale o no del pico	**7 [inflorescencias anormales en especies monoespigadas]**
31	Pico visiblemente curvado respecto al cuerpo del utrículo, tan largo o más largo que éste	***C. porrecta***
–	Pico recto o ligeramente curvado, más corto que el cuerpo del utrículo	**32**
32	Glumas de color marrón a marrón purpúreo, que contrastan con los utrículos; utrículos de 1,5–3 mm; plantas de los departamentos septentrionales del Perú	***C. sodiroi***
–	Glumas de color marrón pálido, estraminosas o hialinas, que no contrastan con los utrículos; utrículos de 2–3,5 mm; plantas del centro y sur del Perú	***C. beckii***
33	Espiga(s) más distal(es) enteramente estaminada(s) y las laterales enteramente pistiladas o andróginas (con flores pistiladas proximales y unas pocas flores estaminadas distales), o espiga(s) más distal(es) andrógina(s) y las laterales pistiladas	**34**
–	Espigas ± similares en apariencia, todas andróginas (con flores pistiladas proximales y algunas flores estaminadas distales), o inflorescencia con todas las espigas unisexuales	**46**
34	Estigmas 2	**35**
–	Estigmas 3	**40**
35	Espigas cilíndricas, fácilmente diferenciables unas de otras, que en su conjunto dan a la inflorescencia un aspecto racemiforme; glumas con ápice romo, de color negro purpúreo a marrón oscuro; tallos bien desarrollados o plantas acaulescentes	**36**
–	Espigas cortamente oblongas, superpuestas unas con otras y a menudo no fácilmente distinguibles entre ellas, dando a la inflorescencia un aspecto espiciforme o subcapitado; glumas con ápice romo, agudo o mucronado, de color marrón rojizo pálido, pajizas o hialinas; plantas acaulescentes, las espigas a menudo ocultas por las hojas	**37**
36	Plantas con tallos cortos, <15 cm de longitud, a menudo plantas acaulescentes; al menos algunas glumas de la parte media de la espiga con un nervio central pálido y conspicuo de 0,2–0,4 mm de ancho; utrículos con los nervios que sobrepasan conspicuamente la mitad de la longitud del utrículo	***C. brehmeri***
–	Plantas con tallos bien desarrollados, generalmente de más de 20 cm de longitud; glumas de la parte media de la espiga con un nervio central pálido estrecho, de 0,1–0,2 mm de ancho; utrículos sin nervios o con nervios débiles, que suelen desaparecer antes de alcanzar la mitad de la longitud del utrículo	***C. enneastachya***
37	Al menos algunas espigas pedunculadas	***C. hypsipedos* p.p**.
–	Todas las espigas sésiles, ocultas por las hojas	**38**
38	Utrículos oblongos, de 4–8 mm de longitud, de color verde amarillento a marrón amarillento o amarillo; hojas de menos de 1,5(2) cm de longitud	***C. collumanthus***
–	Utrículos ovados a elípticos, de 3,5–4,6 mm de longitud, de color marrón o marrón rojizo; hojas de 0,5–4 cm de longitud	**39**
39	Glumas estaminadas de color marrón rojizo, de ápice obtuso; hojas de 1,4 mm de ancho o menos	*C. ruthsatzae* * p.p.
–	Glumas estaminadas de color pajizo, lanceoladas, con el ápice acuminado; hojas de 1,2–3,5 mm de ancho	***C. humahuacaensis***
40	Utrículos papilosos; hojas visiblemente verde azuladas	**41**
–	Superficie del utrículo lisa; hojas de color verde	**42**
41	Tallos generalmente >15 cm de longitud; utrículos fusiformes; hojas canaliculadas	***C. livida***
–	Plantas acaulescentes, si los tallos están desarrollados, entonces <15 cm de longitud; utrículos maduros inflados; hojas planas	***C. brachycalama***
–	Hojas <2,5 mm de ancho; utrículos 2,4–3,3, constreñidos en el ápice en un pico truncado o bidentado; planta acaulescente o casi	***C. punicola* p.p**.
42	Hojas frecuentemente >3 mm de ancho; utrículos de 3–6 mm de longitud, constreñidos en el ápice en un pico ± bífido; plantas acaulescentes o con tallos bien desarrollados	**43**
43	Espigas ovoides a cortamente oblongas, generalmente cada una con <20 utrículos; plantas acaulescentes o con tallos bien desarrollados	**44**
–	Espigas cilíndricas, generalmente cada una con >50 utrículos; plantas con tallos bien desarrollados, generalmente de >30 cm de longitud	**45**
44	Hojas coriáceas, la superficie adaxial de la porción media lisa, irregularmente tuberculada y/o muy diminuta y escasamente escábrida; plantas acaulescentes o casi, las inflorescencias subsésiles, mucho más cortas que las hojas, a menudo ocultas por ellas	***C. paramorum***
–	Hojas herbáceas a subcoriáceas, la superficie adaxial de la porción media escábrida; plantas con tallos bien desarrollados, inflorescencias más cortas, iguales o más largas que las hojas	***C. tamana***
45	Glumas hialinas, de color pajizo, marrón pálido o anaranjado; utrículos de 4–6 mm de longitud	***C. polysticha* p.p**.
–	Glumas de color marrón rojizo oscuro a purpúreo; utrículos de 3–4 mm de longitud	***C. acutata* p.p**.
46	Todas las espigas sésiles o subsésiles, separadas por entrenudos cortos y usualmente superpuestas, dando a la inflorescencia un aspecto de espiga densa; glumas múticas o con el nervio medio continuado en un mucrón exserto de <0,6 mm de longitud	**47**
–	Espigas y/o inflorescencia con al menos algunos pedúnculos bien desarrollados [difíciles de observar en plantas acaulescentes] y entrenudos a menudo visibles, las espigas de último orden superpuestas o no sobre las ramas, de modo que la inflorescencia en conjunto es espiciforme, racemosa o paniculada; glumas múticas, mucronadas o con una arista de hasta 1,6 mm de longitud	**59**
47	Tallos floridos cortos, la inflorescencia sésil o incluso enterrada entre las hojas	**48**
–	Tallos floridos más o menos desarrollados, la inflorescencia sobresaliendo por encima de las hojas	**50**
48	Láminas foliares planas, de 1,2–4,5 mm de ancho; glumas de color pajizo	***C. humahuacaensis* p.p**.
–	Láminas foliares filiformes o de márgenes incurvos, de 0,5–1,5(2,5) mm de ancho; la mayoría de los utrículos son más largos que las glumas, las glumas de color marrón a marrón rojizo; inflorescencia globosa, piramidal o anchamente ovada	**49**
49	Inflorescencia sin brácteas o con brácteas glumáceas que abrazan las espigas inferiores, pero no a la inflorescencia en su conjunto; utrículos sin nervios o débilmente nervados en la base	***C. melanocystis* p.p**.
–	Inflorescencia con una bráctea setácea que la abraza su base, generalmente más larga que la inflorescencia; utrículos visiblemente nervados	*C. ruthsatzae* * p.p.
50	Inflorescencia paniculada, con al menos algunas ramas laterales bien desarrolladas	**51**
–	Inflorescencia capitada o en forma de espiga, congesta, sin ramas laterales evidentes	**53**
51	Glumas sin márgenes hialinos o con éstos muy estrechos	***C. david-smithii* p.p**.
–	Glumas con márgenes hialinos bien desarrollados, al menos >0,2 mm en la mitad distal de la gluma	**52**
52	Glumas pistiladas rojizas a rojizo-purpúreas, ovadas a obovadas, de 1,5–1,9 mm de ancho; utrículos de color pálido, generalmente moteados de rojo, aquenios invaginados lateralmente o asimétricos	***C. catamarcensis* p.p**.
–	Glumas pistiladas negro-purpúreas, lanceoladas, de 1,0–1,4 mm de ancho; utrículos negruzcos en la mitad superior, sin manchas o con pocas manchas negruzcas hacia la base; aquenios no invaginados, simétricos.	***C. pachacutecii* p.p**.
53	Todas las flores son estaminadas o pistiladas	***C. gayana* p.p**.
–	Flores estaminadas y pistiladas dentro de una misma inflorescencia	**54**
54	Al menos la cara adaxial de los utrículos verrugosa o nítidamente arrugada	**55**
–	Cara adaxial de los utrículos lisa, ni verrugosa ni arrugada	**56**
55	Longitud del pico del utrículo menos de la mitad de la del cuerpo	***C. guaguarum***
–	Longitud del pico del utrículo subigual o más larga que la del cuerpo	***C. ros-desertum***
56	Utrículos ≤ 3 mm de longitud, marcadamente ovados, con su anchura máxima en la base o inmediatamente encima de ella, sin nervios o con nervios solo en la cara abaxial y ceñidos a su mitad inferior; pico <1,2 mm de longitud, inciso en su cara abaxial, la incisión a menudo extendida más allá del pico y penetrando en el cuerpo del utrículo	**12. *C. gayana* p.p**.
–	Sin la anterior combinación de caracteres	**57**
57	Cara adaxial de los utrículos con (3)4–10 nervios elevados que recorren la mayor parte de la longitud del cuerpo del utrículo	***C. ownbeyi***
–	Cara adaxial de los utrículos sin nervios o con hasta tres nervios tenues	**58**
58	La mayoría de los utrículos son más largos que las glumas, las glumas de color marrón oscuro; láminas foliares filiformes, de 0,5–1,5(2,5) mm de ancho; inflorescencia globosa, piramidal o anchamente ovada, a menudo más corta que las láminas foliares	***C. melanocystis***
–	La mayoría de los utrículos son o tan largos o más cortos que las glumas, las glumas hialinas a marrón claro; láminas foliares filiformes a planas, generalmente >1,5 mm de ancho; inflorescencia globosa, elíptica u oblonga, usualmente sobrepasando las láminas foliares	***C. ecuadorica***
59	Glumes blackish, purplish or dark reddish-brown, rarely hyaline; plants with flowering stems well-developed; inflorescences paniculate to racemose - if glumes hyaline, then inflorescence paniculate	**60**
–	Glumas hialinas, verdosas, pajizas o marrón rojizo pálido; plantas con tallos floríferos bien desarrollados a acaulescentes; inflorescencia racemosa a espiciforme	**72**
60	Flores con 2 estigmas	**61**
–	Flores con 3 estigmas	**69**
61	Ramas laterales de la inflorescencia de primer o segundo orden con su porción distal densa y compacta, portando multitud de espigas ovoides a cortamente oblongas, dispuestas muy apretadamente y difíciles de distinguir las unas de las otras	**62**
–	Ramas laterales de la inflorescencia de primer o segundo orden portando espigas ovadas, oblongas o cilíndricas, fáciles de distinguir las unas de las otras	**65**
62	Utrículos atenuados en un pico de aproximadamente 1 mm de longitud, el tercio superior del utrículo escábrido; ramas laterales medias e inferiores de la inflorescencia bastante laxas en su porción proximal, su eje generalmente visible entre las espigas hasta su tercio distal	***C. regina***
–	Utrículo atenuado o constreñido en un pico de <1 mm de longitud, el utrículo típicamente liso, raramente muy laxamente escábrido en el pico; ramas laterales medias e inferiores de la inflorescencia densas, su eje usualmente no visible entre las espigas o solo en su mitad proximal	**63**
63	Glumas sin márgenes hialinos o con éstos muy estrechos	***C. david-smithii* p.p**.
–	Glumas con márgenes hialinos bien desarrollados, al menos >0,2 mm en la mitad distal de la gluma	**64**
64	Glumas pistiladas rojizas a rojizo-purpúreas, rara vez hialinas, ovadas a obovadas, de 1,5–1,9 mm de ancho; utrículos de color pálido, generalmente moteados de rojo, aquenios invaginados lateralmente o asimétricos	***C. catamarcensis* p.p**.
–	Glumas pistiladas negro-purpúreas, lanceoladas, de 1,0–1,4 mm de ancho; utrículos negruzcos en la mitad superior, sin manchas o con pocas manchas negruzcas hacia la base; aquenios no invaginados, simétricos.	***C. pachacutecii* p.p**.
65	Utrículo constreñido en la parte superior en un pico de lados paralelos visiblemente bífido en su ápice, generalmente con los márgenes superiores setulosos, rara vez todos los utrículos completamente lisos; utrículos visiblemente nervados	*C. lemanniana* *
–	Utrículo más o menos atenuado en el pico, el ápice truncado o muy brevemente bidentado, márgenes superiores lisos o ciliolados, raramente laxamente setulosos; utrículos nervados o sin nervios	**66**
66	Márgenes del utrículo densamente ciliolados en su tercio superior	***C. canoi* p.p**.
–	Márgenes del utrículo lisos o, raramente, esparcidamente setulosos	**67**
67	Inflorescencia racemosa, si ± paniculada entonces las ramas proximales surgen individualmente de cada nudo y se ramifican solo una vez; utrículos elípticos a elíptico-obovados	***C. pichinchensis***
–	Inflorescencia en panícula abierta con las ramas proximales ramificadas una o varias veces y con numerosas espigas y/o varios pedúnculos que surgen de un único nudo; utrículos elípticos, suborbiculares u obovados	**68**
68	Glumas pistiladas marrón-rojizas, 2.5–3.9 × 0.7–1.2 mm; pico del utrículo truncado	***C. fecunda***
–	Glumas pistiladas negruzcas, 4.0–5.3 × 0.7–1.6 mm; pico del utrículo cortamente bidentado	***C. perprava***
69	Bráctea proximal visiblemente envainante	***C. hebetata***
–	Bráctea proximal sin vaina	**70**
70	La mayoría de las glumas de la parte media de las espigas con el cuerpo membranáceo que no alcanza el ápice del pico de los utrículos	***C. chordalis***
–	Glumas de la parte media de las espigas principales con el cuerpo membranáceo que alcanza o sobrepasa el ápice del pico de los utrículos	**71**
71	Utrículos estrechamente ovados, estrechamente elípticos u oblongos, de 3–4 × 0,9–1,3(1,5) mm, con nervios elevados, ± atenuados en el pico; aquenios estrechamente elípticos a oblongos	***C. jamesonii***
–	Utrículos obovados a suborbiculares, de 2,3–3,2 × 1,2–1,5 mm, sin nervios o con nervios elevados; aquenios marcadamente obovados	***C. colombiana***
72	Estigmas 2	**73**
–	Estigmas 3	**74**
73	Vainas basales de color marrón pálido a pajizo; estigmas ± sinuosos; tallos de hasta 3 cm de altura; a veces hay espigas subterráneas, completamente ocultas en las vainas basales de los brotes	***C. hypsipedos* p.p**.
–	Vainas basales de color marrón rojizo a purpúreo; estigmas visiblemente curvados hacia atrás; tallos generalmente de más de 3 cm de altura; sin espigas subterráneas	***C. lepida* p.p**.
74	Al menos las espigas proximales péndulas; estigmas ± sinuosos	***C. lapazensis***
–	Todas las espigas erectas; glumas múticas; estigmas visiblemente curvados hacia atrás	**75**
75	Utrículos de 2,7–3,3 mm de longitud, con un pico de menos de 0,3 mm de longitud o sin pico; hojas más anchas de 5–8 mm de ancho, subcoriáceas, de color verde azulado	*C. pachamamae* *
–	Utrículos de 3,6–4 mm de longitud, con un pico de 0,6–1,2 mm de longitud; hojas más anchas de hasta 3,5–4 mm de ancho, herbáceas, verdes	***C. roalsoniana***

## Supplementary Material

XML Treatment for Carex paramorum

## Additional information

### Use of AI

No use of AI was reported.

### Author ORCIDs

Pedro Jiménez-Mejías https://orcid.org/0000-0003-2815-4477

Pablo García Moro https://orcid.org/0000-0002-8331-4771

Andrea A. Camilo https://orcid.org/0009-0008-1409-8914

Piero M. Mazzei https://orcid.org/0000-0001-6610-3350

Marcial Escudero https://orcid.org/0000-0002-2541-5427

Santiago Martin-Bravo https://orcid.org/0000-0003-0626-0770

Asunción Cano https://orcid.org/0000-0002-5759-4650

### Data availability

All of the data that support the findings of this study are available in the main text or Supplementary Information.

## Supplementary materials

Supplementary material 1 Confirmed records of *Carex* species in Peru, from herbarium collections and reliable literature Data type xlsx https://binary.pensoft.net/file/1548775This dataset is made available under the Open Database License (http://opendatacommons.org/licenses/odbl/1.0/). The Open Database License (ODbL) is a license agreement intended to allow users to freely share, modify, and use this Dataset while maintaining this same freedom for others, provided that the original source and author(s) are credited. Pedro Jiménez-Mejías, Pablo García-Moro, Andrea A. Camilo, Piero M. Mazzei, Marcial Escudero, Santiago Martin-Bravo, Asunción Cano

Supplementary material 2 Records of *Carex* species in Peru as confirmed from iNaturalist observations Data type xlsx https://binary.pensoft.net/file/1548776This dataset is made available under the Open Database License (http://opendatacommons.org/licenses/odbl/1.0/). The Open Database License (ODbL) is a license agreement intended to allow users to freely share, modify, and use this Dataset while maintaining this same freedom for others, provided that the original source and author(s) are credited. Pedro Jiménez-Mejías, Pablo García-Moro, Andrea A. Camilo, Piero M. Mazzei, Marcial Escudero, Santiago Martin-Bravo, Asunción Cano
